# Systematic analysis of the Frazzled receptor interactome establishes previously unreported regulators of axon guidance

**DOI:** 10.1242/dev.201636

**Published:** 2023-08-01

**Authors:** Yixin Zang, Greg J. Bashaw

**Affiliations:** Department of Neuroscience, Perelman School of Medicine, University of Pennsylvania, 415 Curie Blvd, Philadelphia, PA, 19104, USA

**Keywords:** Netrin, Frazzled, DCC, COP9, Receptor tyrosine phosphatase, ADAM metalloprotease, Axon guidance

## Abstract

The Netrin receptor Dcc and its *Drosophila* homolog Frazzled play crucial roles in diverse developmental process, including axon guidance. In *Drosophila,* Fra regulates midline axon guidance through a Netrin-dependent and a Netrin-independent pathway. However, what molecules regulate these distinct signaling pathways remain unclear. To identify Fra-interacting proteins, we performed affinity purification mass spectrometry to establish a neuronal-specific Fra interactome. In addition to known interactors of Fra and Dcc, including Netrin and Robo1, our screen identified 85 candidate proteins, the majority of which are conserved in humans. Many of these proteins are expressed in the ventral nerve cord, and gene ontology, pathway analysis and biochemical validation identified several previously unreported pathways, including the receptor tyrosine phosphatase Lar, subunits of the COP9 signalosome and Rho-5, a regulator of the metalloprotease Tace. Finally, genetic analysis demonstrates that these genes regulate axon guidance and may define as yet unknown signaling mechanisms for Fra and its vertebrate homolog Dcc. Thus, the Fra interactome represents a resource to guide future functional studies.

## Teaser

**Summary:** An interactome for the conserved axon guidance receptor Frazzled with genetic and biochemical validation of key interaction partners – a valuable resource for future functional studies in both invertebrate and vertebrate systems.

## INTRODUCTION

Dcc is a multifunctional signaling receptor of the immunoglobulin superfamily of transmembrane proteins. Dcc and its *Drosophila* homolog Fra are highly expressed in multiple organ systems, including both the embryonic and adult central nervous system (CNS), the male and female reproductive system, and the heart, the pancreas, the lungs and the gastrointestinal track. The function of Dcc and Fra depends on the cellular context. In the intestinal epithelium, in the absence of binding to its canonical ligand netrin, Dcc is cleaved by caspase 3 and acts as a dependence receptor to activate apoptotic pathways (Forcet et al., 2001). In the CNS, when stimulated by netrin, Dcc and Fra guide axons by modulating the cytoskeleton (Boyer and Gupton, 2018). In *Drosophila* commissural neurons and in the *Drosophila* ovary, Fra functions independently of Netrin to activate gene transcription (Neuhaus-Follini and Bashaw, 2015; Russell et al., 2021; Yang et al., 2009; Zang et al., 2022). Dcc and Fra function in the development and maintenance of diverse tissues and regulate many vital cellular processes, including axon guidance, synaptogenesis, cell migration and cell proliferation (Boyer and Gupton, 2018; Forcet et al., 2001; Horn et al., 2013; Junge et al., 2016; Tu et al., 2015; Zhou et al., 2020). Pathogenic variants in human *DCC* lead to congenital mirror movement disorder, agenesis of the corpus callosum and intellectual disability, and are also implicated in numerous human cancers (Castets et al., 2011; Depienne et al., 2011; Jamuar et al., 2017; Miyake et al., 1994; Shibata et al., 1996; Strohmeyer et al., 1997). Thus, understanding the normal function of Dcc and how disrupting its function leads to adverse effects on human health is an important goal.

Dcc and Fra are best characterized for their roles as axon guidance receptors. During the development of the nervous system, newly differentiated neurons need to extend their axons over long distances through a complex extracellular environment to find their synaptic targets and form functional connections. This dynamic process requires the function of axon guidance receptors, which are transmembrane proteins that are expressed at the tip of navigating axons. Upon binding with their ligands, which are often present as secreted cues or membrane-bound proteins on the surface of neighboring cells, axon guidance receptors activate their downstream signaling effectors to initiate myriad cellular events, including local cytoskeletal remodeling, receptor endocytosis, receptor recycling and degradation, local protein synthesis, and regulation of gene transcription, among many others (Gorla and Bashaw, 2020; Russell and Bashaw, 2018; Zang et al., 2021). Four classical axon guidance receptor-ligand pairs play crucial roles in the formation of neural circuits and have been extensively studied. Yet there is a mismatch between the relatively small number of available guidance receptor-ligand pairs and the overwhelming complexity of neuronal connections in mature nervous systems, which presents a major developmental challenge for all organisms.

One potential solution to this challenge is to repurpose the same guidance cue to elicit divergent responses in different axons that depend on the expression of distinct receptors or receptor combinations. Indeed, this is supported by ample evidence suggesting that the four classical axon guidance cues, netrins, slits, semaphorins and ephrins can all act as bifunctional guidance cues. For example, although netrin is primarily considered to be an attractive cue for its receptor Dcc, it can also induce repulsive responses in axons through the Unc-5 receptor (Finci et al., 2014; Keleman and Dickson, 2001). Furthermore, the Dscam1 receptor can modify the typically repulsive Slit-Robo output into a growth-promoting effect that mediates longitudinal axon growth in the developing *Drosophila* ventral nerve cord (VNC) (Alavi et al., 2016). It is also possible that distinct ligands or interacting proteins modulate the signaling of axon guidance receptors to increase the diversity of signaling outputs. For example, whereas netrin binding to Dcc mediates chemotactic attraction, Draxin binding to Dcc leads to cell contact-dependent axon fasciculation (Liu et al., 2018).

Here, we have focused on uncovering previously unreported regulators of the attractive axon guidance receptor Fra. Although it is well-known that netrin and Dcc promote midline crossing of commissural axons, there is still a vibrant discussion about how these molecules signal to control axon growth and guidance. Although the prevailing view in the field is that Fra and Dcc signal in response to netrin by triggering local cytoskeletal rearrangements in the growth cone, our group and others have identified a ‘non-canonical’ mode of signaling in which Fra and Dcc function at long range by releasing their ICDs, which then translocate to the nucleus to modulate gene transcription (Neuhaus-Follini and Bashaw, 2015; Yang et al., 2009; Zang et al., 2022). However, the mechanism that regulates this Netrin-independent pathway remains an outstanding question. Importantly, we have shown that expression of human DCC in *Drosophila* can substitute for the function of Fra in axon guidance (O'Donnell and Bashaw, 2013). Together with the striking conservation between the fly and human proteins, this observation strongly suggests that Fra and DCC function through conserved binding partners.

In this study, we have used an unbiased approach to identify neuronal-specific Fra-interacting proteins by performing affinity purification coupled with liquid chromatography with tandem mass spectrometry (LC-MS/MS), with protein lysates extracted from *Drosophila* post-mitotic embryonic neurons. By systemically analyzing the Fra interactome, we have uncovered 85 candidate Fra-interacting proteins, including the receptor tyrosine phosphatase Leukocyte-antigen-related-like (Lar), subunits of the constitutive photomorphogenesis 9 (COP9) signalosome (CSN) and the Rhomboid family pseudoprotease Rho-5. Functional studies reveal that these candidate Fra-interacting proteins are enriched in the developing VNC and may play important roles in midline guidance. Our Fra interactome thus represents a valuable resource that should guide future functional studies in neuronal development and many other tissue contexts in both invertebrate and vertebrate systems.

## RESULTS

### Signals from the midline are required for *comm* expression

**Fig. 1.**
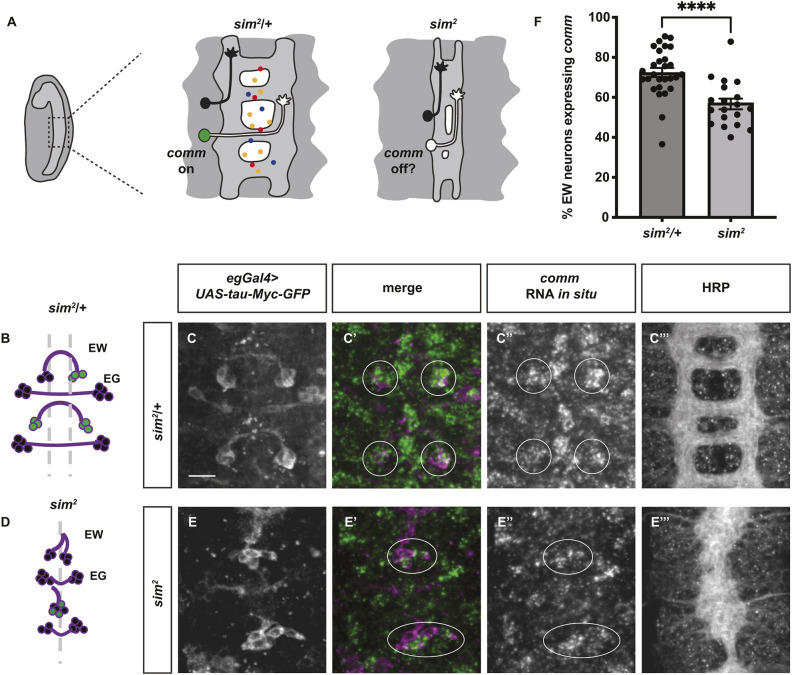
**Signals from the midline are required for *comm* expression.** (A) Schematic diagram showing the effect of *sim* mutants on midline development and the predicted effect on *comm* expression. (B-F) Stage 14 embryos with GFP labeling the eagle neurons (in purple) and *comm* expression shown by RNA FISH (in green). (B-C‴) In heterozygous control embryos, EW neurons form stereotypical commissures and express *comm* in their soma. (D-E‴) In *sim^2^* mutants, the cell bodies and axons of EW neurons are shifted medially and a significant proportion of EW neurons no longer express *comm*. Cell bodies of the EW neurons are outlined. (F) Quantification of the percentage of segments with EW neurons expressing *comm*. Statistical analysis was performed using an unpaired Student's *t*-test. Scale bar: 10 µm. Anterior is upwards.

The midline of the *Drosophila* VNC serves as an important intermediate target for commissural axons, where these axons need to cross the midline before projecting to the contralateral side of the body. Midline glia secrete an array of axon guidance cues, including both the attractive cue Netrin and the repulsive cue Slit. It has been shown that Fra can downregulate Slit-dependent repulsion by functioning as a transcriptional activator to promote the expression of *commissureless* (*comm*) (Neuhaus-Follini and Bashaw, 2015; Yang et al., 2009; Zang et al., 2022)*.* Comm inhibits repulsion by diverting newly synthesized Slit receptor Robo1 to late endosomes for degradation (Keleman et al., 2002, 2005). Importantly, the transcriptional activity of Fra does not depend on Netrin, yet Netrin is the only known Fra ligand in the *Drosophila* system (Neuhaus-Follini and Bashaw, 2015; Yang et al., 2009). Because the midline is an important source of instructive axon guidance signals, we tested whether midline signals are required for *comm* expression. To do this, we used *single-minded* (*sim^2^*) mutants, which lack all midline cell lineages due to the loss of the transcription factor Sim: a master regulator of midline development (Fig. 1A) (Nambu et al., 1990, 1991). To reliably quantify midline crossing events of an easily identifiable subpopulation of commissural neurons, we used *eagle-Gal4* (*egGal4*) to drive the expression of *UAS-tau-Myc-GFP*, which clearly delineates both the cell bodies and the axons of the eagle population of commissural interneurons. Three eagle neurons per hemisegment, termed EW neurons, project axons across the posterior commissure (Fig. 1B). In control embryos, EW cell bodies are positioned on either side of the midline and all EW axons cross the midline (Fig. 1B,C). In *sim^2^* mutants, however, due to the absence of midline cells, the EW cell bodies are shifted medially (Fig. 1D,E). To confirm that the absence of *sim* broadly affects the organization of the entire VNC, rather than affecting just the eagle neurons, we stained all axons in the VNC with the HRP antibody and observed a similar medial shift of axons in *sim^2^* mutants (Fig. 1C‴,E‴). Although most segments still contain a total of six EW neurons, suggesting that EW differentiation or viability are not affected in *sim^2^* mutants, we observe that *comm* expression is significantly reduced in *sim^2^* mutants compared with control embryos (Fig. 1C′,F). Here, *comm* expression is measured by the percentage of EW neurons that express *comm* RNA *in situ* hybridization (RNA FISH) puncta in the soma. This result demonstrates that signals from the midline are required for *comm* expression. The decrease of *comm* expression seen in *sim^2^* mutants closely mirrors what has been reported previously for *fra* and *tace* mutants (Neuhaus-Follini and Bashaw, 2015; Zang et al., 2022), leading us to hypothesize that unidentified midline signals may be required to activate the non-canonical Fra pathway.

### A candidate approach to identify new Fra ligands in *Drosophila*

Next, we employed a candidate approach to try to identify new Fra ligands. Draxin and Cerebellin are alternative ligands for Dcc; however, these proteins are not present in invertebrate systems (Haddick et al., 2014; Islam et al., 2009). Interestingly, *C. elegans* MADD-4 is another conserved and secreted protein that physically interacts with Fra's orthologue UNC-40, to induce an attractive guidance response in muscle arms and sensory neurons (Seetharaman et al., 2011). MADD-4 also controls synaptic localization of GABA receptors through binding and recruiting UNC-40 and neuroligin (Tu et al., 2015; Zhou et al., 2020). Thus, we selected Nolo (*Drosophila* MADD-4) as a candidate Fra ligand and investigated its function in Fra-dependent midline axon guidance.

Nolo belongs to the A Disintegrin and Metalloproteinase with Thrombospondin motifs-like (ADAMTS-like) protein family, which contains ADAMTS ancillary domains but lacks the catalytic domain and proteolytic activity. Nolo is required in glia to control motor functions in larva and adult flies (Meyer et al., 2014), yet its function during embryonic development has not been studied. First, we investigated Nolo expression using RNA FISH in developing *Drosophila* embryos. At stage 14, when the majority of commissural axons are crossing the midline, Nolo is expressed in a distinct subset of cells, some of which are located in close proximity to the trajectory of both EW and EG commissural axons (Fig. S1A-B″). Interestingly, a subset of Nolo-expressing cells are co-labelled with markers for RP motor neurons (Fig. S1C-C‴), which rely on the Fra pathway for the midline crossing of their axons and dendrites, as well as for their innervation of peripheral muscle targets (Labrador et al., 2005; Santiago and Bashaw, 2017). Using a chromosomal deficiency line that covers the *nolo* locus (*nolo^DF^*), we observe that *nolo* mutants show no EW crossing defects (Fig. S2A-C) and no phenotype in HRP-labeled axons (Fig. S2DE). RNA FISH in *nolo* mutants confirms that *nolo* expression is indeed absent from stage 16 embryos (Fig. S1D,D′), yet we also observed maternal RNA in early-stage embryos before the onsite of zygotic transcription (Fig. S1E). It is therefore possible that the absence of phenotype in *nolo* mutants is due to maternal RNA being translated into proteins that are still present in *nolo* zygotic mutants. To bypass this maternal effect, we used the FraΔC-sensitized genetic background and tested whether reducing *nolo* levels could enhance or suppress the FraΔC-dependent midline crossing defects (Fig. S2F-J). In this background, we overexpress a truncated form of the Fra receptor (FraΔC) that has its entire cytoplasmic domain replaced by an HA-tag and that functions as a dominant negative. When FraΔC is expressed in eagle neurons, EW axons fail to cross the midline in ∼30% of segments (Fig. S2F,J) (Garbe et al., 2007). Embryos missing one copy of *nolo* show significant suppression of this FraΔC-dependent non-crossing phenotype, which can be further enhanced when both copies of *nolo* are removed (Fig. S2G-J). This result suggests that Nolo is a negative regulator of midline axon guidance and inhibits midline crossing, which is inconsistent with a role for Nolo in canonical Fra-dependent axon attraction. To study whether Nolo is required for *comm* expression, we scored the percentage of EW neurons expressing *comm* in *nolo* mutants compared with heterozygous sibling controls (Fig. S2K-M). We found no change in *comm* expression, indicating that Nolo does not regulate *comm* transcription. Together, our results suggest that Nolo is unlikely to function with Fra in either the canonical or non-canonical pathway to promote midline crossing of commissural axons.

### An unbiased proteomic screen reveals novel regulators of Fra signaling

**Fig. 2.**
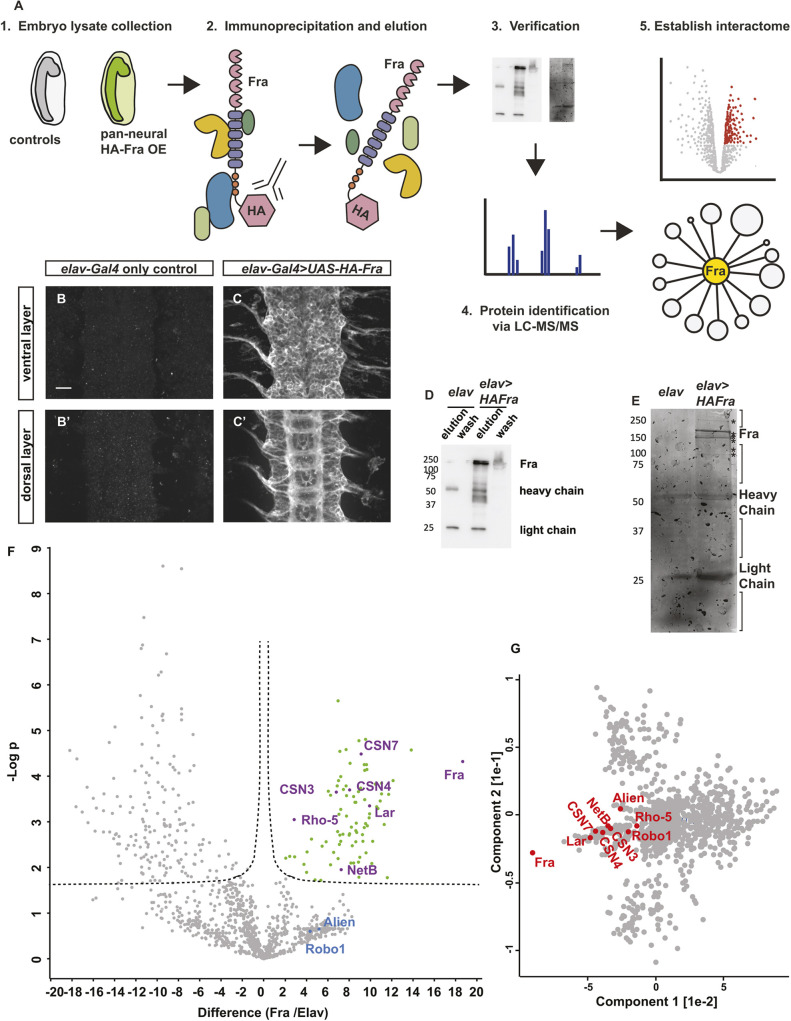
**An unbiased proteomics screen to identify previously unreported Fra-interacting proteins.** (A) Schematic diagram describing the workflow. OE, overexpression. (B-C′) HA staining in stage 14 control embryos (*elav-Gal4* alone) (B,B′) or in embryos that pan-neurally overexpress HA-tagged Fra (C,C′). (B,B′) In control embryos, there is no HA staining. (C) In the ventral layer of the nerve cord, overexpressed Fra is observed on the membrane of all neurons. (C′) In the dorsal layer of the nerve cord, overexpressed Fra is observed on the ladder-like axon tracks. Scale bar: 10 µm. (D,E) Embryonic lysates were collected from control embryos and Fra-expressing embryos. Immunoprecipitation was performed with a HA antibody and is verified by western blotting (D) and silver staining (E). (D) In control immunoprecipitants, no HA-tagged protein is detected. In immunoprecipitants collected from Fra-expressing embryos, Fra is detected at ∼200 kDa. (E) In the silver-stained gel, the band with the highest intensity detected at around 200 kDa likely corresponds to Fra. Many additional bands are detected only in the Fra-overexpression sample, likely corresponding to proteins that interact with Fra, as indicated by the asterisks. (F) A volcano plot depicting the Fra interactome. Proteins that are significantly more abundant in Fra-overexpression samples are shown in green. Fra, Netrin-B and a group of candidate proteins that were selected for follow-up studies are in purple. Robo1 and Alien (in blue) are also more abundantly detected in Fra-overexpression samples compared with controls, but their *P*-values do not reach significance. R0=0.1, FDR=0.05. (G) Principal component analysis of all proteins identified in the Fra interactome. Fra, Netrin-B and Robo1, as well as a group of candidate Fra-interacting proteins that were selected for follow-up studies, are in red. These proteins form a sub-cluster that separates from the main protein cluster in the middle of the graph.

As our candidate approach was not successful, we next performed an unbiased proteomic screen to identify novel Fra interactors through affinity purification coupled with LC-MS/MS (Fig. 2A). Embryonic lysates were prepared from overnight collections of *Drosophila* embryos that pan-neurally overexpress a N-terminally HA-tagged Fra, or from embryos that express only the pan-neural driver *elav-Gal4* as controls. For the negative control condition, we performed two replicates and for the Fra overexpression condition we performed three replicates (subsequently referred to as Elav1, Elav2 for the control samples and Fra1, Fra2 and Fra3 for the Fra-overexpression experimental samples), with each replicate containing embryos collected from the same parental population of flies on different days. We verified the expression of HA-tagged Fra with immunostaining (Fig. 2B-C′). Fra and proteins that physically interact with Fra were immunoprecipitated using an HA antibody, then eluted with an acidic glycine elution buffer (pH=2.0). To verify our pull-down approach, we performed western blots with an HA antibody, and we observed the expected Fra band at around 200 kDa (Fig. 2D). Silver staining revealed that additional bands exist in the Fra-overexpression condition and not in the control condition, which likely correspond to additional Fra-interacting proteins that are present in the immunoprecipitants from Fra-overexpression conditions (Fig. 2E).

Label-free LC-MS/MS analysis on the immunoprecipitants yielded a list of identified peptides. Conventionally, a protein with a fold change higher than two is considered differentially enriched in mass spectrometry datasets. First, we noted that Netrin-B (NetB), the canonical ligand of Fra, and Robo1, a known interactor with Fra, are enriched in the Fra interactome with high fold-change, demonstrating the specificity of our Fra interactome (Fig. S3A). To characterize the quality of our dataset, we generated histograms showing the distributions of protein abundance and observed that all five samples follow normal distributions (Fig. S3B). Density scatter plots show that protein abundance is generally well-correlated between replicates, as indicated by the Pearson correlation coefficients (r) that are larger than 0.8 (Fig. S3C). Protein abundance is not well correlated when comparing control samples with Fra-overexpression samples, clearly indicating that the two experimental groups contain different proteomes (Fig. S3D). Principal component analysis and hierarchical clustering analysis on the five samples display the expected clustering pattern, where the experimental condition is the primary component separating the controls from the experimental samples (Fig. S4A,B). The top two protein clusters in the vertical dendrogram represent proteins that are less detected in Fra-overexpression samples compared with controls, and the biological significance of these proteins is unclear (Fig. S4B,C). The third largest protein cluster contains proteins that are more abundant in Fra-overexpression samples and are detected at lower levels in one out of the two control samples (Fig. S4D). The fourth largest cluster, which includes both Fra and Netrin, contains proteins that are more abundant in Fra-overexpression samples compared with controls, corresponding to potential Fra interactors (Fig. S4E,F).

Next, we generated a volcano plot showing 86 proteins that are significantly more abundant when Fra is overexpressed compared with controls, including both Fra and Netrin (Fig. 2F). After excluding Fra from the list (Table S1), we refer to the remaining 85 proteins as candidate Fra-interacting proteins. Interesting candidates include the receptor tyrosine phosphatase Lar, CSN subunits (CSN7, CSN4 and CSN3) and the Rhomboid pseudoprotease Rho-5. One other CSN subunit, Alien, is also more abundantly detected in Fra-overexpression samples compared with control samples, with a high iBAQ fold-change (Fra/Elav) of 36.4. The adjusted *P*-value for Alien does not reach significance levels because the variability within the Fra replicates is too high. Nevertheless, Alien is still an interesting candidate worthy of further consideration. The same applies to Robo1, which is known to interact with Dcc (Bai et al., 2011), highlighting the fact that a non-significant *P*-value does not necessarily exclude the possibility of interaction. As variability in mass spectrometry samples is common due to biological and technical issues, we used principal component analysis as another measure to visualize proteins that are differentially detected between experimental groups (Fig. 2G). We observed that the majority of proteins cluster together in the middle of the graph, most likely representing proteins with similar protein abundance profiles in all five samples, and thus they cannot be separated from other proteins based on experimental conditions. Outlier proteins form additional clusters because they exhibit biological properties that separate them from the main cluster. Indeed, candidate Fra-interacting proteins identified in the volcano plot belong to one sub-cluster, indicating that proteins from this sub-cluster likely represent differentially detected proteins (Fig. 2G).

**Fig. 3.**
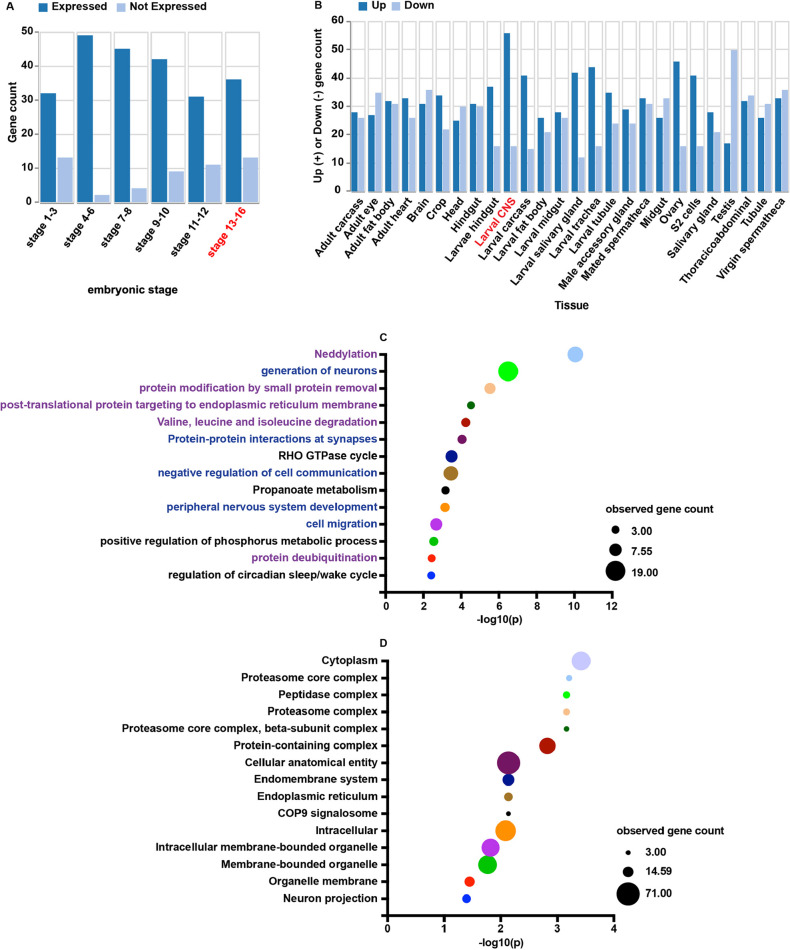
**Gene ontology study on candidate Fra-interacting proteins.** (A,B) GO analysis on the expression patterns of candidate Fra-interacting proteins indicates that they are most abundantly expressed in stage 13-16 embryos (A) and larval CNS (B), indicated in red. (C,D) GO analysis of the biological processes (C) and the cellular components (D) that are enriched in candidate Fra-interacting proteins. Biological processes related to the development or function of neurons are in blue, whereas biological processes related to post-translational modification of proteins are in purple. The size of the circle representing a GO category represents the number of genes observed in that category.

**Fig. 4.**
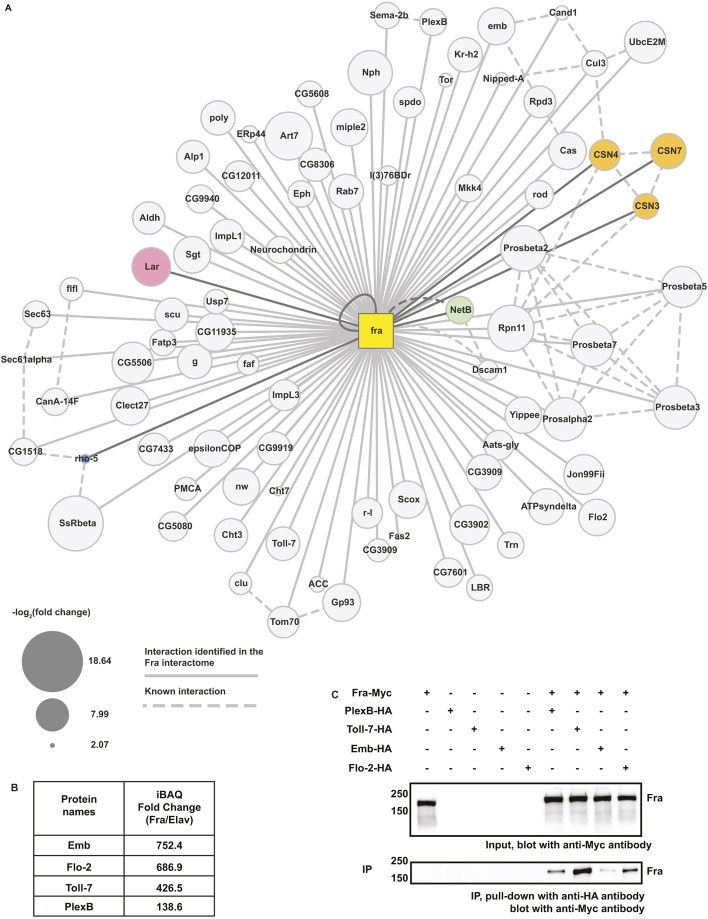
**Network enrichment study and biochemical validation.** (A) A stick and ball diagram representing the Fra interactome. The yellow square in the middle of the diagram represents Fra and the circles at the periphery represent the 85 Fra interacting proteins (NetB plus 84 previously unknown interacting proteins) identified in this study. Solid lines connecting Fra and interacting proteins represent interactions identified for the first time in this study, whereas dashed lines represent known interactions that have been experimentally determined in the past. Circles representing Lar, CSNs and Rho-5, which were selected for functional analysis, and the known Fra ligand NetB, are differently colored and the lines representing their interaction with Fra are highlighted in dark gray. The size of the circles represents the value of -log2[iBAQ fold change (Fra/Elav)] for each Fra-interacting protein. (B) A table summarizing the iBAQ fold change (Fra/Elav) of the four candidate Fra-interacting proteins tested in the immunoprecipitation assay, including Emb, Flo-2, Toll-7 and PlexB. (C) Cell lysates were collected from *Drosophila* S2R^+^ cells transiently transfected with the indicated constructs. Immunoprecipitation was performed with an anti-HA antibody. Fra co-immunoprecipitates with all four candidate Fra-interacting proteins were tested.

As expected, Gene Ontology (GO) analysis of the spatial and temporal expression profiles of candidate Fra-interacting proteins in *Drosophila* revealed that they are expressed throughout embryogenesis, including stage 13-16, and are enriched in the larval CNS (Fig. 3A,B). GO analysis of the biological process of these proteins revealed that terms associated with the development or function of neurons are enriched, further demonstrating the specificity and biological relevance of our Fra interactome (Table S2; Fig. 3C). Additionally, biological processes related to post-translational modification of proteins, such as neddylation and ubiquitylation, are enriched, potentially representing previously unreported pathways that regulate the level or activity of Fra (Table S2; Fig. 3C). The Fra interactome is enriched for proteins with known intracellular localization, with the proteosome complex, the endoplasmic reticulum and the COP9 signalosome as the highest enriched categories (Table S3; Fig. 3D). We represented the Fra interactome with a ‘ball and stick’ diagram, with Fra in the middle and connections with all 85 candidate Fra-interaction proteins (Fig. 4A). In this diagram, interactions predicted by our Fra interactome are shown with solid lines, whereas previously known physical interactions between proteins are shown with dashed lines. Because proteins included in our Fra interactome could either bind to Fra directly or indirectly, by forming a complex with Fra and other proteins, we expected to see networks of proteins enriched in the Fra interactome. Indeed, several protein networks are present among candidate Fra-interacting proteins, including the COP9 signalosome (CSN3, CSN4 and CSN7), the proteosome (Prosalpha2, Prosbeta2, Prosbeta3, Prosbeta5, Prosbeta7 and Rpn11), protein processing machinery in the endoplasmic reticulum (SsRbeta, rho-5, CG1518, Sec61alpha and Sec63) and the Cullin3-RING ubiquitin ligase (Cul3, Cand1, UbcE2M and CSN4) (Fig. 4A).

To verify interactions, we performed co-immunoprecipitation (co-IP) in *Drosophila* S2R^+^ cells with tagged proteins. We selected candidate Fra-interacting proteins with a wide range of iBAQ fold-changes (Fra/Elav) to test the validity of both high-confidence and low-confidence candidate Fra-interacting proteins (Fig. 4B). Selected proteins include: (1) Embargoed (Emb), which encodes an exportin involved in protein export from the nucleus; (2) Flotillin 2 (Flo2), a scaffolding protein involved with secretory pathways; (3) Toll-7, a transmembrane Toll receptor previously shown to be involved in axon and dendrite targeting in the *Drosophila* olfactory system (McIlroy et al., 2013; Ward et al., 2015); and (4) Plexin B (PlexB), which mediates repulsive axon guidance by interacting with semaphorins. Additionally, all four proteins are expressed in the developing *Drosophila* VNC (Collier et al., 2000; Kambris et al., 2002) (the Berkeley Drosophila Genome Project). To perform co-IP, S2R^+^ cells were transiently transfected with plasmids encoding HA-tagged candidate Fra-interacting proteins and Myc-tagged Fra, and pull-down was performed with a HA antibody. We observed that all four proteins are readily immunoprecipitated with Fra, demonstrating that they are true Fra interactors (Fig. 4C).

### Lar, CSN subunits and Rho-5 are candidate Fra-interacting proteins

**Fig. 5.**
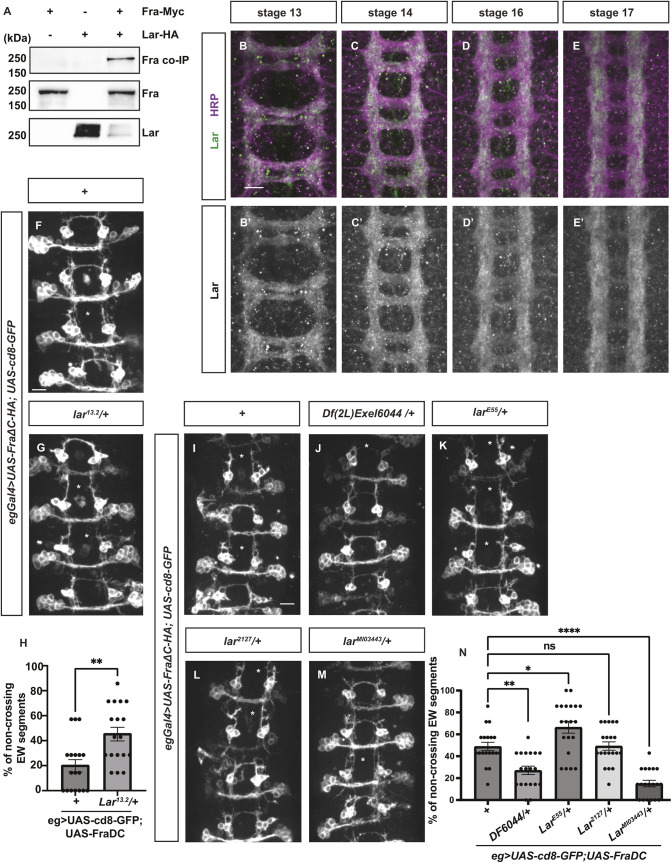
**The receptor tyrosine phosphatase Lar is a candidate Fra-interacting protein.** (A) Cell lysates were collected from *Drosophila* S2R^+^ cells transiently transfected with the indicated constructs. Immunoprecipitation was performed with an anti-HA antibody. Fra co-immunoprecipitates with Lar. (B-E′) Lar expression is shown in *Drosophila* embryos at different developmental stages. Lar antibody staining is shown in green and HRP staining is shown in purple. In pre-crossing stages (stage 13 and 14), Lar is expressed on both longitudinal axons and the commissures. In post-crossing stages (stage 16 and 17), Lar expression on the commissures is notably decreased, whereas Lar expression on longitudinal axon tracks remains largely unchanged. (F-H) Stage 16 *Drosophila* embryos with GFP labeling the eagle neurons. (F) In this experiment, 20% of EW axons fail to cross the midline in embryos overexpressing FraΔC alone in eagle neurons. (G) Further removing one copy of *lar* leads to a significant increase in non-crossing defects in EW axons. Asterisks indicate segments with EW axon non-crossing defects. (H) Quantification of the percentage of segments with non-crossing EW axons. Statistical analysis was performed with one-way ANOVA. (I-N) Stage 16 *Drosophila* embryos with GFP labeling the eagle neurons. (I) In this experiment, 40% of EW axons fail to cross the midline in embryos overexpressing FraΔC alone in eagle neurons. (J-N) Further removing one copy of *lar* leads to different effect on the percentage of non-crossing defects in EW axons, which is quantified in N. Asterisks indicate segments with EW axon non-crossing defects. Statistical analysis was performed with one-way ANOVA.

To examine the functional relevance of the Fra interactome, we selected several candidate Fra-interacting proteins to test their role in midline axon guidance. First, we focused on the receptor tyrosine phosphatase Lar, which encodes a transmembrane protein that binds to the Heparan Sulfate Proteoglycan Syndecan (Sdc) and is required for motor axon guidance (Fox and Zinn, 2005; Krueger et al., 1996). Importantly, binding assays performed with Lar ECD-AP on live dissected *Drosophila* embryos that are mutant for *sdc* still retain prominent AP staining on the axon scaffold, indicating that additional Lar-interacting proteins are localized to axons (Fox and Zinn, 2005). This result suggests that Fra could potentially interact with Lar. By overexpressing tagged Fra and Lar in the *Drosophila* S2R+ cell lines and performing co-immunoprecipitation between the two proteins, we first confirmed that Fra and Lar physically interact (Fig. 5A). Next, we examined the expression pattern of Lar. Antibody staining revealed that Lar is highly expressed in CNS axons (Fig. 5B-E′). In pre-crossing and crossing stages (stage 13 and stage 14), Lar is highly expressed in both longitudinal axon tracks and the commissures (Fig. 5B-C′). Notably, in post-crossing stages (stage 16 and stage 17), Lar expression remains high on longitudinal axons but is greatly diminished on the commissures (Fig. 5D-E′). This expression pattern correlates with the expression pattern of Comm, the transcriptional target of Fra, which is restricted to pre-crossing commissural neurons (Keleman et al., 2002). Next, we used the FraΔC genetic background to investigate the function of Lar in midline crossing, incorporating multiple *lar* alleles to ensure thorough and rigorous testing. We included the *Df(2L)Exel6044* line, which contains a chromosomal deletion that covers the *lar* locus; the *lar^13.2^* line, which contains a nonsense mutation in the extracellular FNIII-8 domain of Lar (Krueger et al., 1996); the *lar^2127^* line, which contains a nonsense mutation in the extracellular FNIII-6 domain of Lar (Maurel-Zaffran et al., 2001); the *larE55* line (also known as Df(2L)E55), which contains a chromosomal deletion that covers part of Lar extracellular domains (Krueger et al., 1996); and the *lar^MI03443^* line, which contains a coding intronic MiMIC insertion in the extracellular portions of the *lar* locus (Venken et al., 2011). Interestingly, although one copy of the *lar^13.2^* or the *lar^E55^* allele enhances the FraΔC-dependent non-crossing phenotype, the *Df(2L)Exel6044* and the *lar^MI03443^* lines show suppression, and the *lar^2127^* line has no effect (Fig. 5F-N). This result indicates that different truncations or manipulations of the Lar protein have distinct impacts on midline axon guidance, suggesting that Lar is important for this process. However, the precise mechanism underlying the involvement of Lar remains unclear. Unexpectedly, homozygous *lar^13.2^* mutant embryos show severe midline guidance defects, where we observed almost complete disruptions of commissure formation with 100% penetrance (Fig. S5A to S5B′, S5G). Additionally, in these embryos, cells in the nerve cord exhibit enlarged and irregular shapes with punctate membranes (Fig. S5B′,B″). These phenotypes likely result from a background mutation, because *lar^13.2^/lar^2127^* and *lar^13.2^/lar^E55^* compound heterozygotes show normal axon scaffolds. The same phenotypes are also observed in *fra^3^ ,lar^13.2^* double mutants that were generated by recombination (Fig. S5F,G), suggesting that this background mutation may be linked to the *lar* locus.

**Fig. 6.**
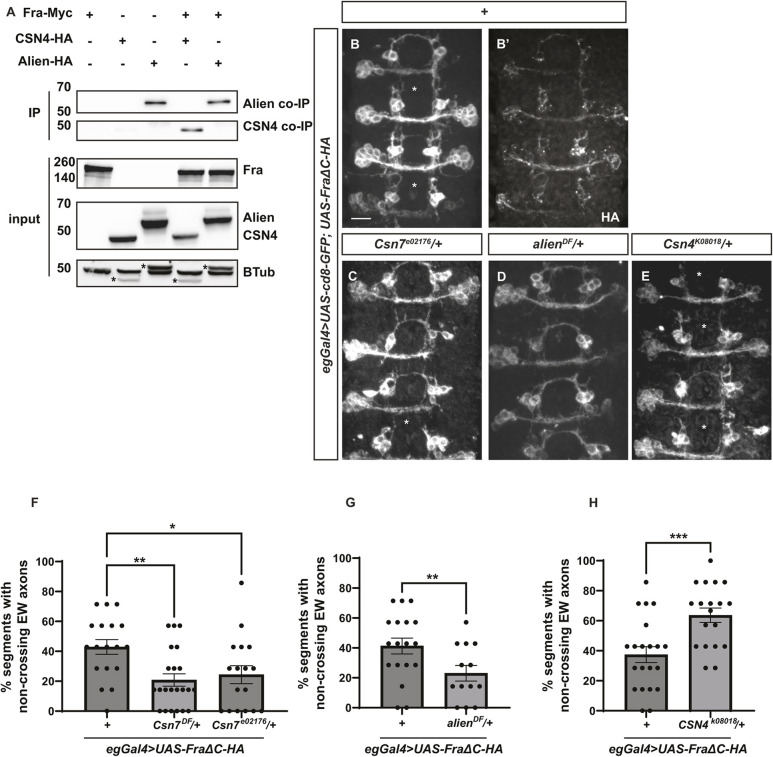
**CSN subunits are candidate Fra-interacting proteins that function in midline axon guidance.** (A) Cell lysates were collected from *Drosophila* S2R^+^ cells transiently transfected with the indicated constructs. Immunoprecipitation was performed with an anti-Myc antibody. CSN4 co-immunoprecipitates with Fra, while Alien shows non-specific binding. Non-specific bands in the tubulin loading control are indicated by an asterisk. (B-E) Stage 16 *Drosophila* embryos with GFP labeling the eagle neurons. (B,B′) In embryos that overexpress the HA-tagged FraΔC receptor, around 40% of the EW axons fail to cross the midline. HA staining is shown in B′. (C,D) When one copy of *Csn7* (C) or *alien* (D) is removed, more EW axons cross the midline. (E) When one copy of *Csn4* is removed, fewer EW axons cross the midline. Asterisks indicate segments with EW axon non-crossing defects. (F-H) Quantifications of the percentages of segments with non-crossing EW axons in embryos with the indicated genotypes. Scale bar: 10 µm. Anterior is up. Statistical analyses were performed with one-way ANOVA in F and an unpaired Student's *t*-test in G and H.

The COP9 signalosome (CSN) is a conserved multi-subunit protein complex that affects various cellular processes, including protein degradation, cell cycle regulation, stem cell self-renewal and differentiation, receptor signaling activities, dendritic arborization, and embryogenesis (Huang et al., 2014; Knowles et al., 2009; Oron et al., 2002, 2007; Pan et al., 2014; Singer et al., 2014; Suh et al., 2002). CSN is a master regulator of the cellular ubiquitylation and neddylation levels by modulating the activity and assembly of cullin-RING ubiquitin ligases, which is the largest conserved family of E3 ubiquitin ligases responsible for the degradation of ∼20% of the proteins processed by the proteasome (Dubiel et al., 2020). In addition, CSN also controls gene transcription, either by interacting with transcription factors or by directly binding to DNA (Oron et al., 2007; Singer et al., 2014). The multiple subunits of CSN (CSN3, CSN4, CSN7 and Alien) that were identified in our Fra interactome thus represent promising candidate Fra-interacting proteins that could potentially function either in the trafficking and degradation of Fra or in its non-canonical activity as a transcription factor. First, we demonstrated that CSN4 physically interacts with Fra (Fig. 6A). In contrast, we noted the presence of co-immunoprecipitated Alien protein even in the absence of Fra expression, indicating potential non-specific binding. Consequently, this precludes the feasibility of conducting interaction analysis between Alien and Fra. Next, using the FraΔC background, we observe significant suppression of the FraΔC-dependent non-crossing phenotype in EW axons when one copy of *Csn7* is removed either using a chromosome deficiency line (*Csn7^DF^*) or a P-element mediated mutant line (*Csn7^e02176^*) (Fig. 6B-F). A similar suppression is observed when we remove one copy of *alien* with a deficiency line (*alien^DF^*, Fig. 6D,G). However, reducing the dose of *Csn4* with a P-element mediated mutant line (*Csn4^k08018^*) leads to the opposite phenotype where a significantly higher percentage of EW axons fail to cross the midline compared with the FraΔC background alone (Fig. 6E,H). These results suggest that although CSN7 and Alien are negative regulators of midline crossing, CSN4 facilitates midline crossing. It is unclear whether these divergent functions of CSN subunits in midline axon guidance are CSN complex dependent or independent.

**Fig. 7.**
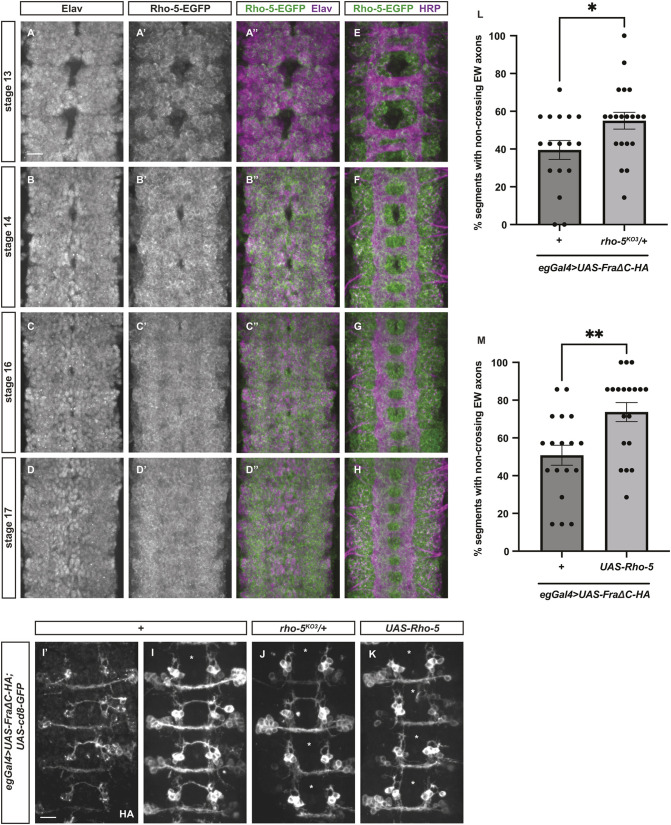
**Rho-5 is expressed in the developing *Drosophila* VNC and functions as a positive regulator of midline crossing.** (A-H) In *Drosophila* embryos of the indicated developmental stages, Rho-5 protein expression is detected by the endogenous GFP-tag (green). (A-D″) All neurons in the VNC are labeled with the neuronal marker Elav, which is shown in purple. Across all developmental stages, Rho-5 is detected in a large subset of neurons. (E-H) HRP labels all axons in the VNC (purple). Across all developmental stages, Rho-5 is not detected in the axon tracks. (I-M) Stage 16 *Drosophila* embryos with GFP labeling the eagle neurons. (I) 40% of EW axons fail to cross the midline in embryos overexpressing HA-tagged FraΔC alone in eagle neurons. HA staining shown in I′. (J) Further removing one copy of *rho-5* leads to a significant increase of non-crossing defects in EW axons, which is quantified in L. (K) Overexpressing Rho-5 also leads to a significant increase of non-crossing defects in EW axons, which is quantified in M. Asterisks indicate segments with EW axon non-crossing defects. Statistical analyses were performed with an unpaired Student's *t*-test. Scale bar: 10 µm. Anterior is up.

Rho-5 belongs to the Rhomboid family of pseudoproteases and is the sole *Drosophila* orthologue of the vertebrate iRhom proteins (Freeman, 2014). In *Drosophila*, Rho-5 participates in ER-associated degradation (ERAD) to regulate the secretion of the Spitz ligands of the epidermal growth factor receptor (Zettl et al., 2011). In mammalian cell culture, iRhom1 acts as a stimulator of proteosome activity (Lee et al., 2015). Importantly, in vertebrates, iRhom2 is required for the trafficking and maturation of ADAM17 through the secretory pathway, and controls stimulated ADAM17 activity on the plasma membrane (Cavadas et al., 2017). We have recently demonstrated that Tace and its mammalian orthologue ADAM17 are key regulators of Fra and Dcc signaling during commissural axon guidance (Zang et al., 2022). Based on this evidence, we believe Rho-5 is an interesting candidate that could function either in the trafficking and degradation of Fra or to regulate the activity of Tace. To better understand the function of Rho-5, we examined its expression pattern in the developing *Drosophila* VNC (Fig. 7A-H). We generated an endogenously GFP-tagged Rho-5 fly line (Rho-5-EGFP). Using Elav as a marker for CNS neurons and HRP as a marker for CNS axons, we observe that Rho-5 is expressed in the soma of a large subset of Elav-positive neurons but is not expressed on the axon tracks (Fig. 7A-H). This expression is very similar to the expression pattern and localization of Tace (Zang et al., 2022), suggesting that Rho-5 could potentially interact with Fra alone or form a complex with both Tace and Fra to regulate the proteolytic cleavage of Fra by Tace. Next, we tested the function of Rho-5 using the FraΔC background. Surprisingly, either reducing or increasing the levels of Rho-5 leads to a significant enhancement of FraΔC-dependent non-crossing defects in EW axons (Fig. 7I-M). This result suggests that Rho-5 is a positive regulator of midline crossing, yet overexpressing Rho-5 also has a gain-of-function effect. Because Rho-5 controls the maturation and activity of many substrates, it is possible that overexpressing Rho-5 affects the function of another protein that is essential for midline axon guidance, with Tace as a likely candidate.

## DISCUSSION

Axon guidance receptors are essential for the establishment of functional neural circuits, and disruptions in their functions are causatively linked with multiple neurodevelopmental disorders in humans (Engle, 2010; Nugent et al., 2012). These proteins also play essential roles in the development and function of other organ systems and are implicated in multiple types of cancer (Laws and Bashaw, 2022). Here, we have focused on the attractive axon guidance receptor Fra in *Drosophila* and have established a high-quality Fra interactome through an unbiased affinity purification mass spectrometry approach. Eighty-five candidate Fra-interacting proteins were identified, most of which were previously not known to function in axon guidance at the midline, including Lar, CSN subunits and Rho-5 (Table S1). Functional characterization of these candidates demonstrates that they exhibit specific expression patterns in the developing VNC and function in regulating midline axon guidance. Together, the list of candidate Fra-interacting proteins represents a valuable resource for furthering our understanding of proteins that regulate axon guidance functions. Many of the pathways enriched in the Fra interactome, such as protein modification and maturation, are likely to also function outside the nervous system. Thus, we believe these proteins could also lead us towards unifying mechanisms that control Fra expression and activity in other tissue contexts. Finally, because the majority of candidate Fra-interacting proteins are also conserved in humans, these proteins hold the potential of revealing novel drug targets for cancer and other diseases.

### Establishing a Fra interactome

To maximize the preservation of physiologically relevant protein interactions, we employed an unbiased affinity purification mass spectrometry approach on samples collected from developing *Drosophila* neurons *in vivo*. Using the *Elav-Gal4* pan-neural driver, we expressed tagged Fra in all neurons in the *Drosophila* embryo, which still constitutes just a small fraction of the total cells. Fortunately, collection of *Drosophila* embryonic lysates is easily scalable, and we were able to obtain sufficient amounts of biological material for the subsequent immunoprecipitation and mass spectrometry analysis by collecting embryos from large populations of flies (∼2000 flies per replicate). In total, 85 candidate Fra-interacting proteins are enriched in the Fra interactome, including Netrin, the canonical ligand of Fra, and the DCC-interacting protein Robo1. Because interaction between secreted ligands and their receptors are generally weak and transient, the enrichment of Netrin in the Fra interactome demonstrates that our method of detection is sensitive and biologically relevant. GO analysis indicates that candidate Fra-interacting proteins are enriched for pathways including protein trafficking and maturation, post-translational modification (neddylation, ubiquitylation, deubiquitylation and dephosphorylation) and protein degradation (Table S2). It is likely that proteins in these categories contribute to previously unreported regulatory mechanisms that control the level, activity or function of Fra. Notably, proteins with known nuclear function, such as the exportin Emb and several transcription factors or co-factors are enriched in the Fra interactome. It is tempting to speculate that these proteins function in the non-canonical pathway to mediate or regulate the transcriptional activity of Fra. Finally, candidate Fra-interacting proteins include several proteins with known functions in axon guidance, such as Sema-2b, PlexB, Eph and Dscam1. It remains to be explored when and how they interact with Fra, and what is the biological relevance of such interactions.

One potential caveat of pan-neurally overexpressing Fra is the possibility that this manipulation will change the levels of transcription and translation of many genes and proteins. This is based on previous observations that characterized Fra as a transcription factor that regulates the expression of *comm* and likely other genes as well. One could argue that these putative Fra-induced genes will be enriched in Fra-overexpression samples, and, as a result, would more likely to be retained by immunoprecipitation either by non-specifically binding to the HA antibody or to beads, or by forming complexes with other Fra-interacting proteins. To argue against this possibility, we noted that the overall protein abundance between experimental conditions is comparable, as shown by the distribution histograms (Fig. S3B), suggesting that Fra-overexpression does not induce global changes in protein translation. Additionally, we selected both high-confidence and low-confidence candidate Fra-interacting proteins, and confirmed that they indeed physically interact with Fra (Fig. 4B,C), supporting the validity of our results.

### Phosphatase-dependent regulation of Fra/Dcc signaling

Notably, we identified two classes of protein phosphatases among candidate Fra-interacting proteins: the receptor tyrosine phosphatase Lar and subunits of the serine/threonine phosphatases. The latter includes CanA-14F, which encodes one of the catalytic subunits of calcineurin, and Falafel, which encodes the regulatory 3 subunit of protein phosphatase 4. It is unclear whether Fra is a direct substrate of these phosphatases, or whether Fra recruits phosphatases to dephosphorylate other proteins to regulate midline crossing. Here, we focused on Lar, which physically interacts with Fra. Lar is transiently enriched in the commissures during crossing. Moreover, our results in the FraΔC genetic background suggest that Lar is important for midline crossing. It has been shown that tyrosine phosphorylation in the cytoplasmic domain of Fra is dispensable for receptor activity, as Fra variants that have all nine cytoplasmic tyrosines mutated to phenylalanines are able to fully rescue midline crossing and muscle targeting phenotypes in *fra* mutants (O'Donnell and Bashaw, 2013). Thus, it is unlikely that Lar directly de-phosphorylates Fra to regulate midline crossing. Indeed, it has been shown in the *Drosophila* larval visual system that Lar can function independently of its phosphatase activity in the targeting of R7 photoreceptor axons in the medulla and in the targeting of R1-6 photoreceptor axons in the lamina (Hofmeyer and Treisman, 2009; Prakash et al., 2009). If Lar does not require catalytic activity for its function in midline axon guidance, it is tempting to speculate that Lar can act as a co-receptor for Fra to regulate Fra signaling. A similar mechanism is observed in the *Drosophila* larval hematopoietic niche, where Lar physically associates with Insulin-like receptor to inhibit insulin signaling and is required for niche maintenance (Kaur et al., 2019). Structure-function analysis in the future that dissects the requirement of Lar extracellular domains will help us assess the involvement of its ligand in midline crossing.

### Subunits of the COP9 signalosome as candidate Fra-interacting proteins

Our Fra interactome includes several subunits of the COP9 signalosome (CSN). CSN subunits are implicated in neurodevelopmental disorders and neurodegenerative diseases (Djagaeva and Doronkin, 2009a,b), suggesting that they could have neuronal specific functions. CSN is best known as the deneddylase for cullin-RING ubiquitin ligases (CRLs), but it is also capable of removing Nedd8 from non-cullin proteins (Kandala et al., 2014). Could Fra be a substrate for neddylation and deneddylation, and what could be the biological significance of neddylating Fra? Gene ontology enrichment analysis of these newly discovered neddylated proteins identified with mass spectrometry suggests that neddylation controls both gene transcription and protein translation, as well as the regulation of actin cytoskeleton (Lobato-Gil et al., 2021). Because Fra and/or Dcc is involved in all three of these processes, neddylating Fra could potentially be important for its canonical or non-canonical functions, or both. Furthermore, by comparing the relative abundance of neddylated proteins that are enriched in the nucleus and the cytosol, another recent proteomics study suggests that neddylation promotes protein translocation between the nucleus and the cytoplasm (Li et al., 2020). Thus, it is also possible that CSN subunits facilitate the nuclear translocation of Fra. Finally, Fra might interact with CSN subunits to regulate the function of CRLs. As a result, Fra could control ubiquitin-mediated degradation of many CRL substrates and profoundly alter the proteome. Cand1, which functions by sequestering and stabilizing unneddylated CRLs in *Drosophila* (Kim et al., 2010), was also enriched in our Fra interactome as a candidate Fra-interacting protein. It is thus possible that Fra acts as the bridge that brings CSN and Cand1 into close proximity, so that Cand1 can rapidly bind to CSN-deneddlyated CRLs to inhibit their function. In this study, we have shown that CSN7 and Alien inhibit midline crossing, whereas CSN4 promotes midline crossing (Fig. 6). CSN4 shows the opposite phenotype of the other two subunits, suggesting that it might function independently of the CSN complex. Indeed, this is supported by the observation that the transcriptome of *Csn4* mutants is distinct from *Csn5* mutants (the catalytic subunit of CSN) (Oron et al., 2007).

### Insights into the regulation of Fra and Dcc function by proteolytic processing

The ADAM metalloprotease Tace and its vertebrate homologue ADAM17 are required for midline crossing of commissural axons and they function by cleaving both Fra and Dcc to regulate their signaling properties (Zang et al., 2022). Notably, we identified Rho-5, the sole *Drosophila* homolog of the vertebrate iRhom proteins, as a candidate Fra-interacting protein in our proteomic screen. The iRhom proteins are essential for the maturation and activity of ADAM17 (Adrain et al., 2012; Cavadas et al., 2017). Here, we have shown that Rho-5 exhibits overlapping expression with Tace, as both are specifically enriched on the soma of VNC neurons (Fig. 7) (Zang et al., 2022). We have also shown that *rho-5* phenocopies *tace* in the FraΔC background and that both are required for midline crossing (Zang et al., 2022). Furthermore, overexpression of Rho-5 inhibits midline crossing, which could result from overexpressed Rho-5 binding and quenching the activity of Tace. Based on this observation, it is tempting to hypothesize that Fra might bind to Rho-5 to promote the disassociation of Tace from Rho-5, which may trigger the proteolytic activity of Tace and the ensuing non-canonical Fra signaling.

In conclusion, we systematically characterized the Fra interactome and established candidate Fra-interacting proteins that regulate axon guidance at the midline. The Fra interactome facilitates our understanding of the regulation and function of Fra, by revealing many candidates that potentially act in different aspects of Fra signaling. Furthermore, 13 out of the 85 candidate Fra-interacting proteins are unannotated proteins whose functions are yet to be studied in the *Drosophila* system. We believe that our Fra interactome serves as a valuable resource that can guide functional studies of candidate proteins in the future, which undoubtedly will provide us with new insights into how neural circuits are established during development. Furthermore, our study represents a cell-type specific and temporally controlled approach to establish the interactome of a specific transmembrane protein in intact tissues, which could be broadly applied to other molecules of interest and cell or tissue types of interest.

## MATERIALS AND METHODS

### *Drosophila* stocks

The following *Drosophila* mutant alleles were used in this study: *sim^2^* [Bloomington Drosophila Stock Center (BDSC) 2055], *nolo^DF^* [*Df(2L)ED1466*, BDSC 9340], *lar^13.2^* (BDSC 8774), *lar^E55^* (BDSC 3076), *Df(2L)Exel6044* (BDSC 7516), *lar^MI03443^* (BDSC 40760), *lar^2127^* (BDSC 63796), *Csn7^e02176^* (BDSC 18023), *Csn7^DF^* [*Df(2R)Exel6058*, BDSC 7540], *Csn4^k08018^* (BDSC 10765), *alien^DF^* [*Df(2L)Exel6021*, BDSC 7505) and *rho-5^KO3^* (a gift from Dr Matthew Freeman, University of Oxford, UK). The following Gal4 lines were used in this study: *eagle-Gal4* and *elav-Gal4.* The following transgenic lines were used in this study: *P{UAS-FraΔC-HA}* (Garbe et al., 2007), *P{10UAS-HA-Fra}* (Neuhaus-Follini and Bashaw, 2015) and *P{UAS-Rho-5}* (a gift from Dr. Matthew Freeman)*.* The following endogenously tagged line was used in this study: *Rho-5-EGFP.* All crosses were carried out at 25°C.

### Molecular cloning

The gene sequence of *toll7* does not contain any introns. Thus, to generate the p10UAST-Toll7-3xHA plasmid, we subcloned the entire coding sequence of Toll7 from genomic DNA extracted from a single *w^1118^* male fly, with the Toll7_XhoI_fwd (TCACTCGAGATGGCGGCAATCCTGCTG) and Toll7_KpnI_rev (GTCGGTACCCACCAGATACGCCTGAACATGG) primer set, and the XhoI (R0146, NEB) and KpnI-HF (R3142, NEB) restriction enzymes into the empty p10UAST-3xHA vector.

To generate the p10UAST-PlexB-3xHA plasmid, we subcloned the entire coding sequence of PlexB from the RE22882 clone (DGRC) with the PlexB_EcoRI_fwd (GCGGGAATTCATGTTGCGAAAGGAATTGTATT) and the PlexB_KpnI_rev (GCTGGTAcCGCAATTAGATGTGCAATCACC) primer set, and the EcoRI- HF (R3101, NEB) and KpnI-HF (R3142, NEB) restriction enzymes into the empty p10UAST-3xHA vector.

To generate the p10UAST-Emb-3xHA plasmid, we subcloned the entire coding sequence of Emb from the LD45706 clone (DGRC) with the Emb_NotI_fwd (ATTAGCGGCCGCATGGCGACAATGTTGACATC) and the Emb_KpnI_rev (TATCGGTACCTTCGTCCTGCATATCCTCGG) primer set, and the NotI-HF (R3189, NEB) and KpnI-HF (R3142, NEB) restriction enzymes into the empty p10UAST-3xHA vector.

To generate the p10UAST-Flo-2-3xHA plasmid, we subcloned the entire coding sequence of Flo-2 from the RE74011 clone (DGRC) with the Flo2_EcoRI_fwd (CGGGAATTCATGGGCAACATACACACGACGG) and the Flo2_XhoI _rev (TATCTCGAGCGCCTTGGCCCCCGGTATCT) primer set, and the EcoRI-HF (R3101, NEB) and XhoI (R0146, NEB) restriction enzymes into the empty p10UAST-3xHA vector.

To generate the p10UAST-Lar-3xHA plasmid, we subcloned the entire coding sequence of Lar from a Lar cDNA clone (gift from Dr. Kai Zinn) with the Lar_EcoRI_fwd (CTGGAATTCATGGGTCTGCAGATGACAGC) and the Lar_KpnI _rev (GCGCGGGTACCGTTTGTATAATTGTCGAATGAGCCC) primer set, and the EcoRI-HF (R3101, NEB) and KpnI-HF (R3142, NEB) restriction enzymes into the empty p10UAST-3xHA vector.

To generate the p10UAST-CSN4-3xHA plasmid, we subcloned the entire coding sequence of CSN4 from the GH09439 clone (DGRC) with the CSN4_XhoI_fwd (GTGACTCGAGATGGCCGCAAACTACGGC) and the CSN4_KpnI _rev (TCGTGGTACCGTTCAGGTTATCCATCCAATCGGG) primer set, and the XhoI (R0146, NEB) and KpnI-HF (R3142, NEB) restriction enzymes into the empty p10UAST-3xHA vector.

To generate the p10UAST-Alien-3xHA plasmid, we subcloned the entire coding sequence of Alien from the LD10463 clone (DGRC) with the Alien_XhoI_fwd (GTGACTCGAGATGTCCGACAACGATGAT) and the Alien_KpnI _rev (TCGTGGTACCGGCCATTTTCTGGACCACAGCGA) primer set, and the XhoI (R0146, NEB) and KpnI-HF (R3142, NEB) restriction enzymes into the empty p10UAST-3xHA vector.

### *Drosophila* S2R^+^ cell culture and transfection

*Drosophila* S2R^+^ cells were cultured at 25°C in Schneider's *Drosophila* medium (21720024, Life Technologies) supplemented with 10% (v/v) FBS (10437-028, Gibco) and 1% (v/v) penicillin and streptomycin (10378-016, Invitrogen). Transient transfections of *Drosophila* S2R^+^ cells were performed according to the manufacturer's recommendations (Effectene Transfection Reagent, 301425, Qiagen). For each well of *Drosophila* S2R^+^ cells that was plated in a six-well plate, 1 µg each of P10UAST expression plasmids were transfected together with 500 ng of a Cu^2+^ inducible PMT-Gal4 plasmid (1042, DGRC). Expression was induced 24 h after transfection by replacing the media with fresh media containing 0.5 mM CuSO_4_. Cell lysates were collected 24 h after induction.

### Embryo collection, fixation and immunostaining

*Drosophila* embryos were collected from overnight cages, then dechorionated, formaldehyde fixed and methanol devittellinized as described by Bashaw (2010). Briefly, to immunostain *Drosophila* embryos, fixed embryos were incubated with the appropriate primary antibodies overnight at 4°C, then incubated with the appropriate secondary antibodies for 2 h at room temperature and stored in 70% glycerol. Embryos were then dissected with polished Tungsten wire under a dissection scope, mounted in 70% glycerol and imaged with a spinning disk confocal system.

Primary antibodies for immunostaining that were used in this study include: rabbit anti-GFP (1:500, a11122, Invitrogen), mouse anti-FasII (1:50, 1D4, DSHB), mouse anti-HA (1:500, 901502, BioLegend), mouse anti-Lar (1:20, 9D82B3, DSHB), mouse anti-Elav (1:20, 9F8A9, DSHB) and Alexa647 goat anti-HRP (1:500, 123-605-021, Jackson).

### RNA *in situ* hybridization

RNA *in situ* hybridization was performed as described by Labrador et al. (2005). DIG-labeled probe for *nolo* was generated by PCR amplifying the *nolo* cDNA from clone GH19218 (DGRC) using the nolo_insitu_fwd (ATGTCGGTGAACATGAACTGGA) and nolo_insitu_rev (TTAAAACGGTTCAACATCGT) primer pair. DIG labeled *comm* probe was generated as previously described by Yang et al. (2009).

### Affinity purification mass spectrometry

Parent flies were allowed to mate and lay embryos for 24 h. Lysates from ∼200 µl of *Drosophila* embryos were collected by dechorionating the embryos with 50% bleach for 2 min, washing the embryo with ample wash buffer (120 mM NaCl supplemented with 0.1% Triton X-100), rinsing the embryos with ice-cold TBSV buffer [150 mM NaCl ,10 mM Tris (pH 8.0) and 2 mM ortho-vanadate], then transferring the embryos to Dounce Tissue Grinders (DWK Life Sciences) and lysing the embryos in 1 ml lysis buffer [TBSV supplemented with 1% Surfact-AMPS NP40 (85124, ThermoFisher) and 1× complete protease inhibitors (11697498001, Roche)] by manual homogenization. Lysates were precleared with 50 µl of a 50%/50% combination of protein A and G agarose beads (15918-014 and 15920-010, Invitrogen) for 2 h at 4°C, then immunoprecipitated by first incubating with mouse anti-HA antibody (2 µl/ml, 901502, BioLegend) overnight at 4°C, then incubating with 50 µl of a 50%/50% combination of protein A and G agarose beads for 2 h at 4°C. We used excessive amounts of beads and antibodies to prevent saturation of beads or antibodies. Immunoprecipitants were eluded from the beads with 100 µl of 0.1 M (pH 2.0) glycine elution buffer, neutralized with minimal amount of 1 M (pH8.0) Tris-HCl solution, then reduced with BME (1610710, Bio-Rad) and boiled for 5 min at 95°C.

To perform mass spectrometry analysis, lysates were loaded on a 4-12% Tris/Tricine gel (Mini-PROTEAN, BioRad) then run until the dye spread to ∼1 cm. The gel was stained with Colloidal Blue (LC6025, Thermo Fisher) then processed by the Proteomics and Metabolomics Facility at the Wistar Institute. Briefly, the entire protein-containing gel regions were cut and digested with trypsin. The digests were analyzed the by LC-MS/MS on a Q Exactive Plus mass spectrometer. MS/MS spectra generated from the LC-MS/MS runs were searched using full tryptic specificity against the UniProt *D. melanogaster* database using the MaxQuant 1.6.2.3 program. Protein quantification was performed using unique peptides. False discovery rates for protein and peptide identifications were set at 1%. Common contaminants including keratins were removed.

### Gel electrophoresis, western blotting and silver staining

Gel electrophoresis and western blotting were performed according to standard protocols. Primary antibodies used in this study for western blotting include mouse anti-HA (1:1000, 901502, BioLegend) and mouse anti-Myc (1:1000, 9e-10, DSHB). Silver staining was performed according to the manufacturer's instructions (1610449, BioRad). Protein gels and blots were visualized with the ChemiDoc Imaging System (171001401, BioRad).

### Mass spectrometry data analysis

A protein is included in the Fra interactome only if it is identified in at least two of the three replicates, in at least one of the conditions. Because larger proteins will generate more peptides, the intensity output for each identified protein was normalized to the number of theoretical peptides to generate iBAQ (intensity based absolute quantification) intensity, which is used for all subsequent analysis as a measurement of protein abundance. For a given protein included in the Fra interactome, iBAQ fold change (Fra/Elav) is calculated as the ratio between the average iBAQ intensity in the Fra-overexpression samples compared with that of the control samples. A protein with an iBAQ fold-change (Fra/Elav) over two is considered to be more abundantly identified in the Fra-overexpression samples compared with the controls.

The LC-MS/MS dataset contains many proteins that are identified only in Fra-overexpression samples but not in control samples, including the Fra protein itself. As a result, the iBAQ intensity of these proteins in the control samples will be 0, which will interfere with subsequent analysis. Thus, we replaced the 0 s with the dataset minimum iBAQ intensity value divided by 2 (i.e. 986). We then performed a log_2_ transformation to compress the dynamic range of our dataset.

We analyzed the LC-MS/MS data set with the Perseus v2.0.7.0 software to generate distribution histograms, density scatter plots and the volcano plot, and to perform principal component analysis and hierarchical clustering analysis. For the final list of candidate Fra-interacting proteins, *P*-values were calculated using an unpaired Student's *t*-test and the difference was calculated as log2(iBAQ fold change Fra/Elav) (Table S1). Gene ontology analysis was performed with Metascape (Zhou et al., 2019) and the STRING database (Szklarczyk et al., 2021).

### Immunoprecipitation in *Drosophila* S2R^+^ cells

Immunoprecipitation in *Drosophila* S2R^+^ cells was performed as described by Neuhaus-Follini and Bashaw (2015). Briefly, *Drosophila* S2R^+^ cells were plated in six-well plates then transiently transfected with the indicated plasmids. Lysates were collected by lysing the cells with 500 µl lysis buffer [TBSV supplemented with 1% Surfact-AMPS NP40 (85124, ThermoFisher) and 1× complete protease inhibitors (11697498001, Roche)]. Lysates were pre-cleared with 30 µl of a 50%/50% combination of protein A and G agarose beads (15918-014 and 15920-010, Invitrogen) for 20 min at 4°C, then immunoprecipitated by first incubating with mouse anti-HA antibody (1.5 µl/ml, 901502, BioLegend) for 2 h at 4°C, then incubating with 30 µl of a 50%/50% combination of protein A and G agarose beads for 30 min at 4°C. Immunoprecipitants were eluded from the beads with 2× Laemmli Sample Buffer (1610737, BioRad) by boiling for 10 min at 95°C.

### Generation of the endogenously GFP-tagged Rho-5 fly line

The endogenously GFP-tagged *Rho-5-EGFP* fly line was generated using the Double-Header method detailed in Li-Kroeger et al. (2018). Briefly, recombinase-mediated cassette exchange (RMCE) events carried out by the PhiC31 integrase exchanged the attP-flanked CRIMIC cassette from the *TI{CRIMIC.TG4.0}rho-5^CR02355-TG4.0^* line with the attB-flanked GFP-forward Double-Header protein trap cassette. Successful RMCE events were screened using four PCR reactions, using the gene-specific Rho5CRIMIC_fwd (CATCCACCCCAGCGAATTCCA) and Rho5CRIMIC_rev (TTTATTCTCGGAGTGCACCGATGTT) primers, and the two primers flanking the Double-Header cassette from Li-Kroeger et al. (2018).

### Quantification and statistical analysis

Analysis of *Drosophila* nerve cord phenotypes was conducted without knowing the genotype. Statistical analysis was conducted with the GraphPad Prism 9 software. An unpaired Student's *t*-test was used for significance comparison between two groups. To compare between multiple groups, significance was assessed using one-way ANOVA with Sidak tests, with family-wise alpha threshold and confidence level set at 0.05 (95% confidence interval). In all column scatter plots, each dot represents one embryo and error bars indicate the s.e.m. A χ^2^ test was used to assess significance in contingency table analysis. *P*-values are represented as follows: *P*>0.05 (n.s.), *P≤0.05, **P≤0.01, ***P≤0.001, ****P≤0.0001.

Phenotypes were scored using Volocity software. For all phenotypes scored in the embryonic *Drosophila* VNC, A1 to A7 abdominal segments were analyzed. *comm* expression in stage 14 EW neurons was scored as described by Neuhaus-Follini and Bashaw (2015). Briefly, for each embryo, we scored the number of EW neurons that have *comm* RNA FISH fluorescent puncta expressed within the border of somatic membranes, then divided the number with the total number of EW neurons in that embryo to obtain the percentage of EW neurons expressing *comm*. Muscle 6/7 cleft innervation in stage 17 FasII labeled motor axons was scored as described by Santiago and Bashaw (2017). Phenotypes from both sides of the ventral muscle were scored if possible. Midline crossing of EW axon in stage 16 embryos was scored as described by Garbe et al. (2007). A segment was considered non-crossing when the EW axons did not extend from the soma, or extended ipsilaterally, or extended contralaterally but stalled before crossing the midline. Midline crossing phenotypes in HRP-stained axon scaffold are quantified as described by Hernandez-Fleming et al. (2017). A segment was considered to have a ‘thin’ commissure when the thickness of the commissure is visibly thinner than a wild-type commissure. A segment was considered to have a ‘missing’ commissure when the segment is missing either one or both of the anterior or the posterior commissures.

## Supplementary Material

10.1242/develop.201636_sup1 Supplementary information
