# Supplementary material for: Systematic analysis of the Frazzled receptor interactome establishes previously unreported regulators of axon guidance
Source: Development. 2023 Aug 1;150(15):dev201636. doi: 10.1242/dev.201636 (PMC10445734; doi:10.1242/dev.201636)
Supplement: Supplementary information [file develop-150-201636-s1.pdf]

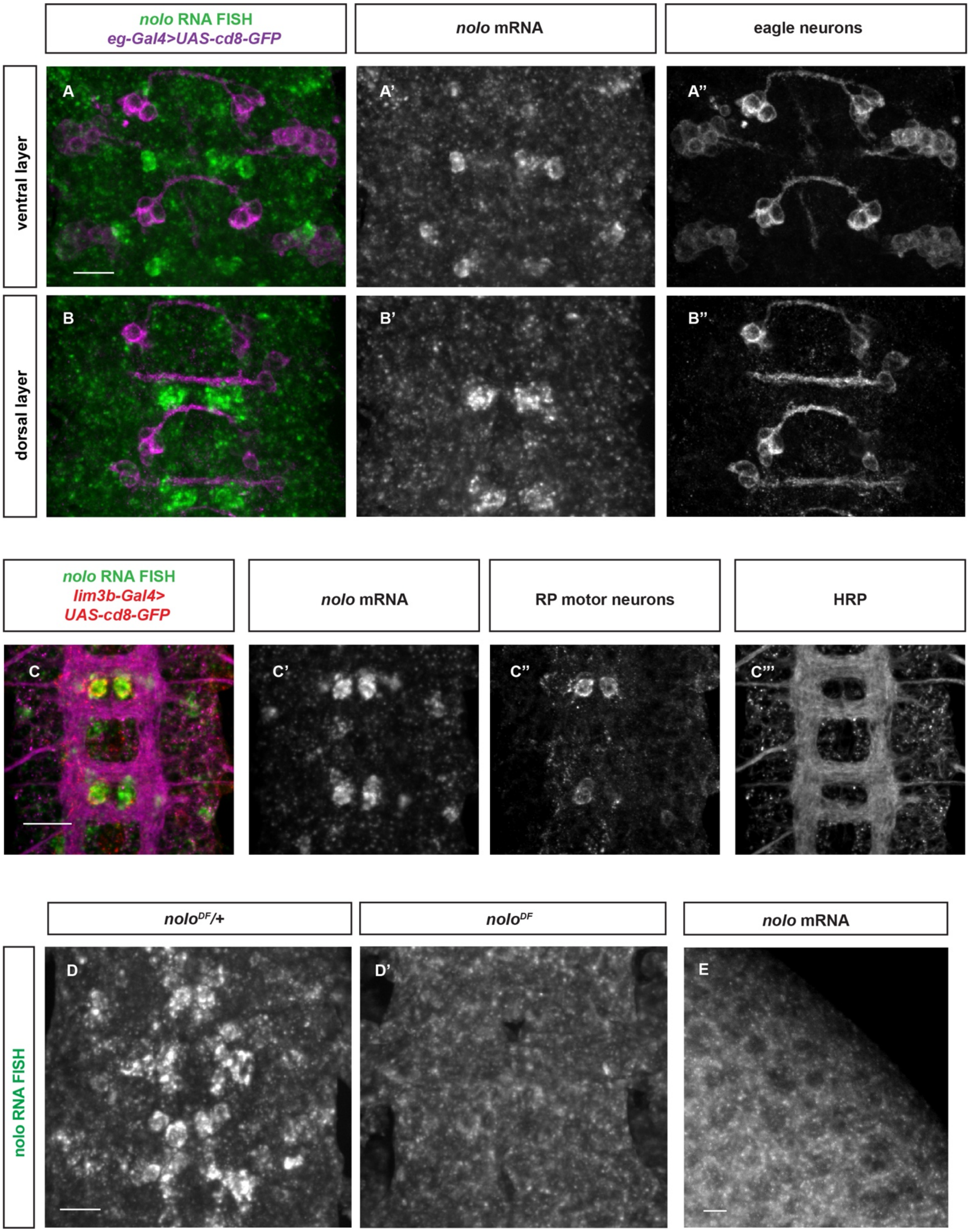

nolo RNA FISH
eg-Gal4>UAS-cd8-GFP
nolo mRNA
eagle neurons
ventral layer
dorsal layer
A
A'
A"
B
B'
B"
nolo RNA FISH
lim3b-Gal4>
UAS-cd8-GFP
nolo mRNA
RP motor neurons
HRP
C
C'
C"
C'''
noloDF/+
noloDF
nolo mRNA
nolo RNA FISH
D
D'
E

Fig. S1. *nolo* is expressed in the developing *Drosophila* VNC

(A to B'') Stage 14 embryos with GFP labeling the eagle neurons (shown in magenta) and *nolo* expression visualized by RNA FISH (shown in green). *nolo* mRNA is expressed in distinct populations of cells in the VNC. *nolo* is not expressed in eagle neurons but is expressed along the trajectory of EW and EG commissural axons. (C to C''') Stage 14 embryos with GFP labeling the RP motor neurons using the *Lim3b-Gal4* driver (shown in red), HRP antibody labeling the axon scaffold (shown in magenta), and *nolo* expression visualized by RNA FISH (shown in green). *nolo* mRNA is expressed in RP motor neurons. (D and D') *nolo* expression visualized by RNA FISH (shown in green) in stage 16 embryos heterozygous for $nolo^{DF}$ (D) or homozygous for $nolo^{DF}$ (D'). (E) Stage 1-2 embryos showing maternally deposited *nolo* mRNA. Scale bars in A to D' and F represent 10μM, scale bar in E represents 40μM.

Development • Supplementary information

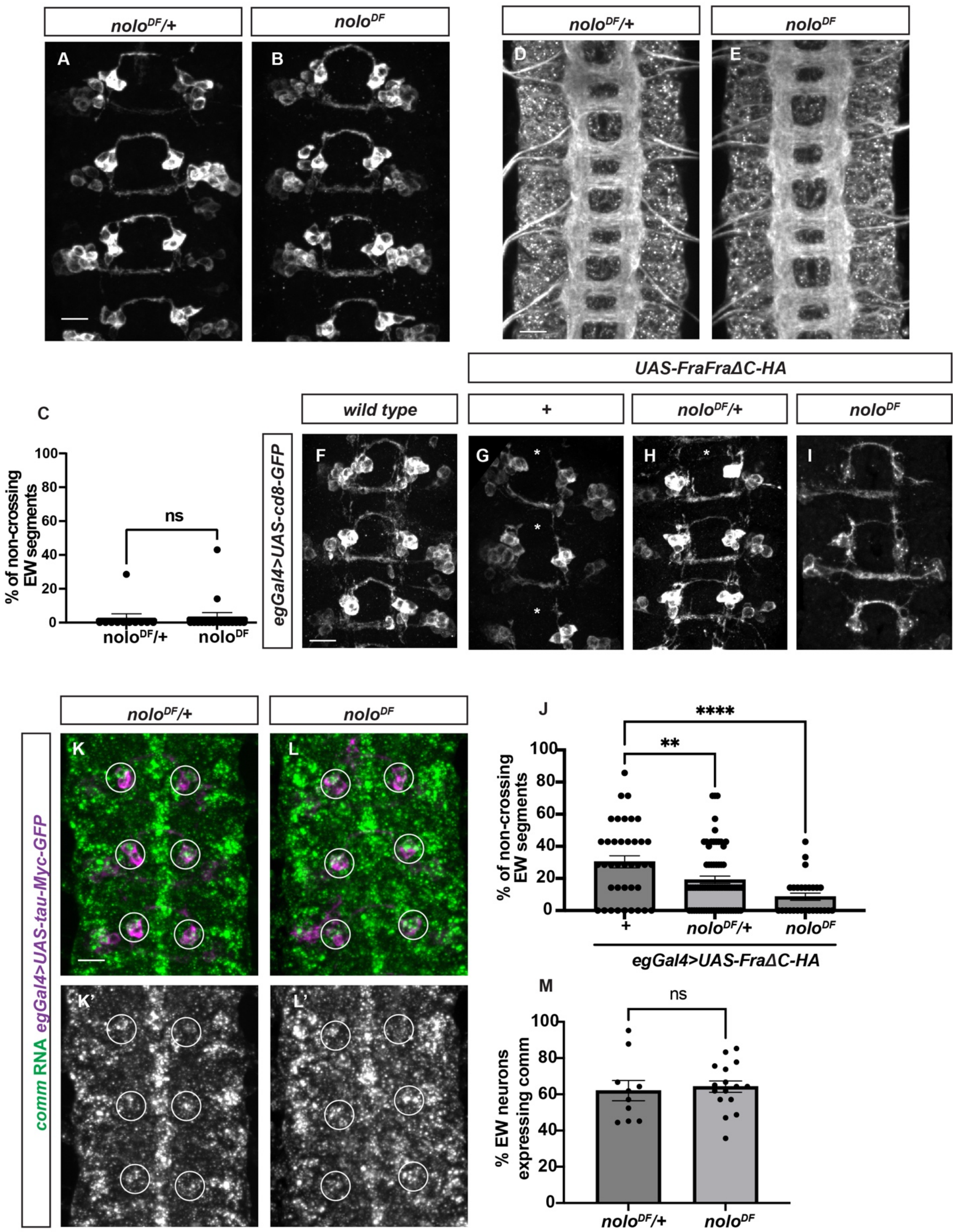

Development • Supplementary information

Fig. S2. *nolo* is unlikely to function in Fra signaling

(A to C) Stage 14 embryos with GFP labeling the eagle neurons. In both sibling control embryos (A) and *nolo* mutant embryos (B), almost all EW axons cross the midline, which is quantified in C. (D and E) Stage 16 embryos will all axons labeled by the HRP antibody. In both sibling control embryos (D) and *nolo* mutant embryos (E), the axon scaffolds show the stereotypical ladder like structure. (F to J) Stage 16 embryos with GFP labeling the eagle neurons in green. (F) In wild-type embryos, all EW axons cross the midline. (G) In embryos that overexpress the FraΔC receptor in eagle neurons, around 30% of the EW axons fail to cross the midline. White triangles indicate segments with EW axon non-crossing defects. (H) When one copy of *nolo* is removed in the FraΔC background, EW axons in more segments cross the midline compared to the FraΔC background alone. (I) When both copies of *nolo* is removed in the FraΔC background, midline crossing of EW axons are largely normal. Asterisks indicate segments with EW axon non-crossing defects. (J) Quantification of the percentage of segments with non-crossing phenotypes in EW axons. (K to M) Stage 14 embryos with GFP labeling the eagle neurons in magenta and *comm* expression shown by RNA FISH in green. (K to L') In both sibling control embryos (K and K') and *nolo* mutant embryos (L and L'), around two thirds EW neurons express *comm.* The cell bodies of EW neurons are outlined with white circles. (M) Quantification of the percentage of EW neurons expressing *comm*. In all images, scale bar represents 10μM. All statistical analyses were performed with Student t-test in C and M, one-way ANOVA in J.

Development • Supplementary information

A

Protein names	iBAQ Fold Change (Fra/Elav)
Fra	409712.1
NetB	153.6
Robo1	20.2

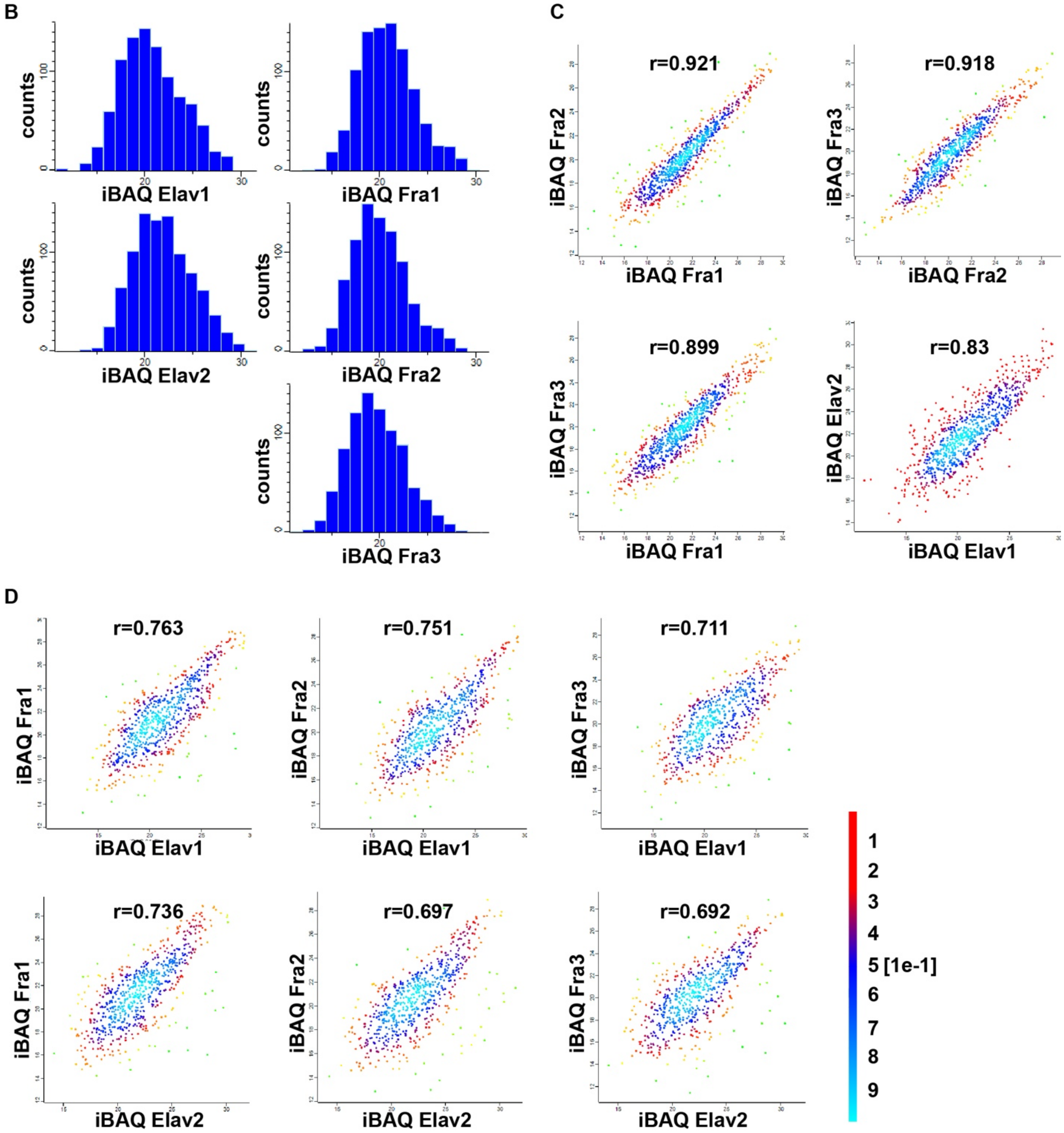

Fig. S3. Characterizing the quality of the proteomics data set with distribution histograms and scatter plots

(A) A table summarizing the iBAQ fold change (Fra/Elav) of Fra, Fra's canonical ligand Netrin-B (NetB), and Fra's known interacting protein Robo1. (B) Histograms showing the distribution of protein abundance in all five samples, which largely follows a normal distribution. (C) Density scatter plots showing the correlation between the three replicates of each experimental condition. All replicates from the same experimental group highly correlate with each other. (D) Density scatter plots showing the correlation between experimental conditions. As expected, the Elav-only control samples do not correlate well with the Fra-overexpression samples. r represents the Pearson correlation coefficient.

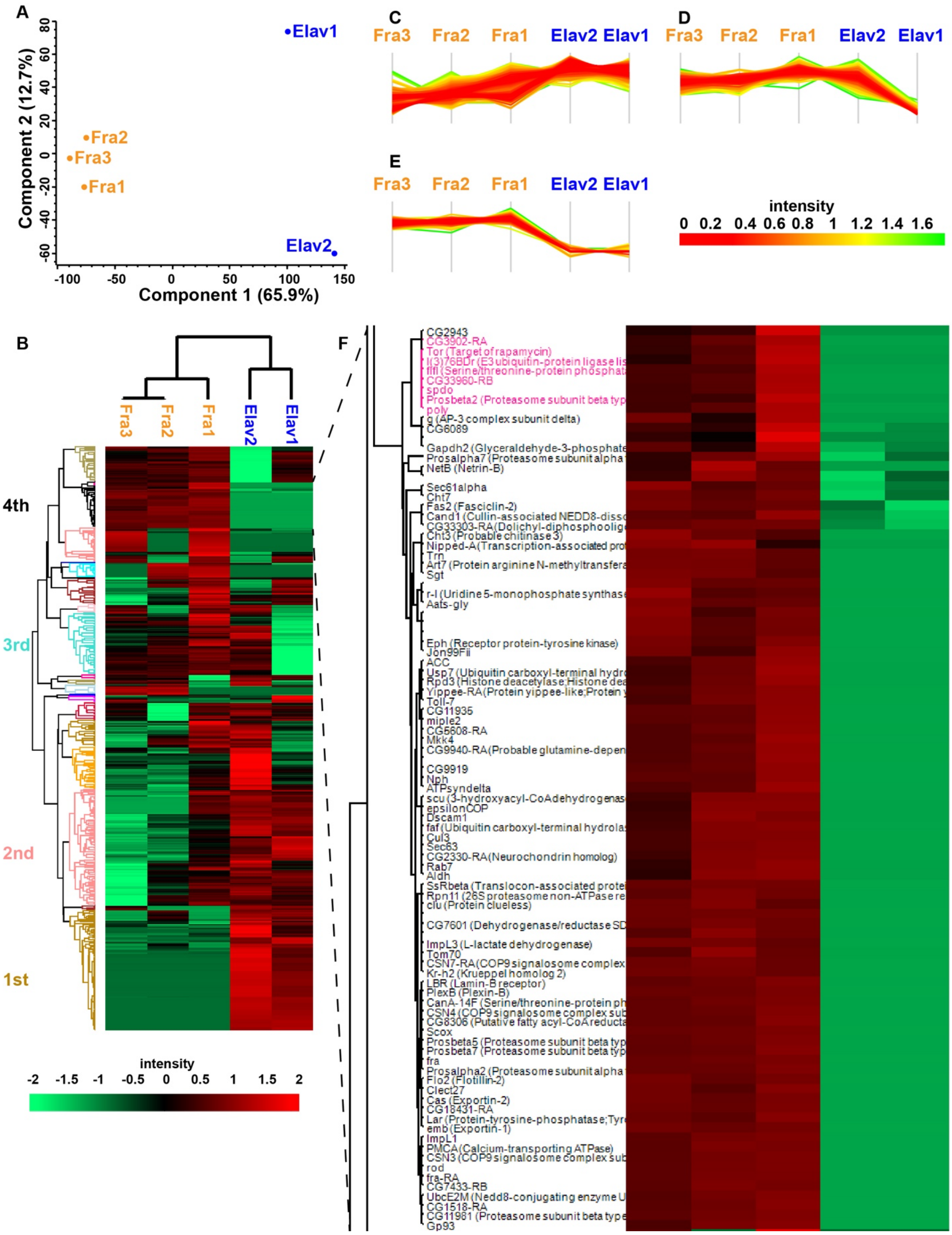
A
Component 2 (12.7%)
Component 1 (65.9%)
Elav1
Elav2
Fra1
Fra2
Fra3
B
4th
3rd
2nd
1st
intensity
-2 -1.5 -1 -0.5 0 0.5 1 1.5 2
C
D
E
Fra3 Fra2 Fra1 Elav2 Elav1
intensity
0 0.2 0.4 0.6 0.8 1 1.2 1.4 1.6
F
CG2943
CG3902-RA
Tor (Target of rapamycin)
l(3)76BDr (E3 ubiquitin-protein ligase lis
fl(fl (Serine/threonine-protein phosphat
CG33960-RB
spdo
Prosbeta2 (Proteasome subunit beta typ
poly
g (AP-3 complex subunit delta)
CG6089
Gapdh2 (Glyceraldehyde-3-phosphate
Prosalpha7 (Proteasome subunit alpha
NetB (Netrin-B)
Sec61alpha
Cht7
Fas2 (Fasciclin-2)
Cand1 (Cullin-associated NEDD8-disso
CG33303-RA(Dolichyl-diphosphooligo
Cht3 (Probable chitinase 3)
Nipped-A(Transcription-associated pro
Trn
Art7 (Protein arginine N-methyltransfera
Sgt
r-l (Uridine 5-monophosphate synthase
Aats-gly
Eph (Receptor protein-tyrosine kinase)
Jon99Fii
ACC
Usp7 (Ubiquitin carboxyl-terminal hydro
Rpd3 (Histone deacetylase;Histone dea
Yippee-RA(Protein yippee-like;Protein y
Toll-7
CG11935
miple2
CG5608-RA
Mkk4
CG9940-RA(Probable glutamine-depen
CG9919
Nph
ATPsyndelta
scu (3-hydroxyacyl-CoA dehydrogenas
epsilonCOP
Dscam1
faf (Ubiquitin carboxyl-terminal hydrolas
Cul3
Sec63
CG2330-RA(Neurochondrin homolog)
Rab7
Aldh
SsRbeta (Translocon-associated prote
Rpn11 (26S proteasome non-ATPase re
clu (Protein clueless)
CG7601 (Dehydrogenase/reductase SD
ImpL3 (L-lactate dehydrogenase)
Tom70
CSN7-RA(COP9 signalosome complex
Kr-h2 (Krueppel homolog 2)
LBR (Lamin-B receptor)
PlexB (Plexin-B)
CanA-14F (Serine/threonine-protein ph
CSN4 (COP9 signalosome complex sub
CG8306 (Putative fatty acyl-CoA reducta
Scox
Prosbeta5 (Proteasome subunit beta typ
Prosbeta7 (Proteasome subunit beta typ
fra
Prosalpha2 (Proteasome subunit alpha
Flo2 (Flotillin-2)
Clect27
Cas (Exportin-2)
CG18431-RA
Lar (Protein-tyrosine-phosphatase;Tyr
emb (Exportin-1)
ImpL1
PMCA(Calcium-transporting ATPase)
CSN3 (COP9 signalosome complex sub
rod
fra-RA
CG7433-RB
UbcE2M (Nedd8-conjugating enzyme U
CG1518-RA
CG11961 (Proteasome subunit beta type
Gp93

Fig. S4. Characterizing the quality of the proteomic data set with hierarchical clustering analysis and principal component analysis

(A) Principal component analysis indicates that all five samples cluster with their respective experimental groups. (B to F) Characterizing the samples with hierarchical clustering analysis and profile plots. (B) Hierarchical clustering analysis performed on all five samples shows that they cluster with their respective experimental groups. Dendrogram on the left side of the heat map represents the clustering pattern of individual proteins included in the data set. (C) Profile plot of the 485 proteins included in the first and second largest protein clusters. These proteins are detected with higher abundance in the Elav control samples compared to the Fra-overexpression samples. (D) Profile plot of the 125 proteins included in the third largest protein cluster. These proteins are more abundant in Fra-overexpression samples compared to the Elav1 but not the Elav2 control sample. (E) Profile plot of the 93 proteins included in the fourth largest protein cluster. These proteins are more abundantly detected in Fra-overexpression samples compared to both control samples and represent potential Fra interacting proteins. This region in the histogram in (B) is enlarged in (F). Both Fra and Netrin-B are identified in this protein cluster.

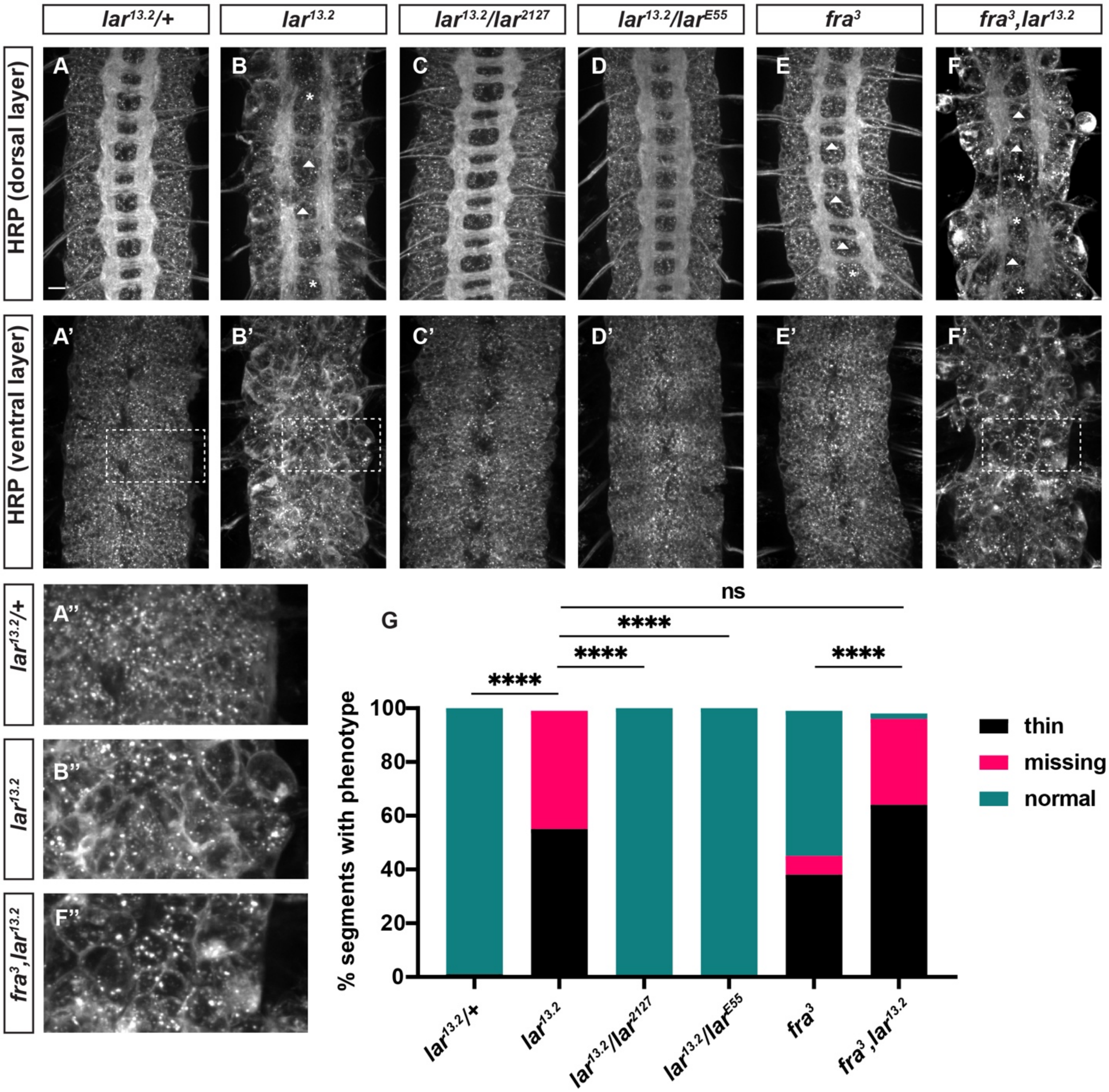
lar13.2/+
lar13.2
lar13.2/lar2127
lar13.2/larE55
fra3
fra3,lar13.2
HRP (dorsal layer)
HRP (ventral layer)
A
B
C
D
E
F
A'
B'
C'
D'
E'
F'
A''
B''
F''
G
ns
****
% segments with phenotype
thin
missing
normal
0
20
40
60
80
100

Fig. S5. *lar*$^{13.2}$ mutants show severe midline crossing defects

(A to F'') Stage 16 *Drosophila* embryos with HRP labeling the axon scaffold. (A and A') heterozygous sibling control embryos show normal axon scaffold and cell morphology. (B and B') *lar*$^{13.2}$ mutants show severe commissure formation deficits, and notable difference in cell morphology. (C to D') Compound heterozygotes show no phenotype. (E and E') commissures are frequently missing or thin in *fra*^{3} mutants. (F and F') *fra*^{3},*lar*$^{13.2}$ double mutant embryos show the same defects as observed in *lar*$^{13.2}$ mutants. Asterisks indicate segments where commissures are missing, arrow heads indicate segments where commissures are thinner than wild type commissures. Regions in dashed lines are enlarged in A'', B'' and F'' to show the cell morphology defect. In all images, scale bar represents 10μM.

(G) Quantification of the percentage of segments that exhibit the indicated phenotype. Statistical analysis was conducted with chi-square analysis, $p<0.0001$. Statistical analyses were also performed by comparing the percentage of normal segments in each genotype using one-way ANOVA, and the significance levels are indicated on the graph. The number of embryos quantified, n=17,18,16,15,16,25.

Development • Supplementary information

Table S1. The Fra interactome.
Mass spectrometry data for 85 candidate Fra interacting proteins, with statistical analysis by student t-test. Difference was calculated as log2(iBAQ fold change Fra/Elav).

Click here to download Table S1

Table S2. GO analysis of the biological process of candidate Fra interacting proteins.
Analysis was performed with Metascape.

Click here to download Table S2

Table S3. GO analysis of the subcellular localization of candidate Fra interacting proteins.
Analysis was performed with the STRING data base.

Click here to download Table S3
