# Supplementary material for: Systematic analysis of the Frazzled receptor interactome establishes previously unreported regulators of axon guidance
Source: Development. 2023 Aug 1;150(15):dev201636. doi: 10.1242/dev.201636 (PMC10445734; doi:10.1242/dev.201636)
Supplement: Supplementary file 1 [file develop-150-201636_review_history.pdf]

Systematic analysis of the Frazzled receptor interactome establishes previously unreported regulators of axon guidance

Yixin Zang and Greg J. Bashaw
DOI: 10.1242/dev.201636

Editor: Francois Guillemot

Review timeline

Original submission:	19 January 2023
Editorial decision:	14 February 2023
First revision received:	16 June 2023
Editorial decision:	5 July 2023
Second revision received:	6 July 2023
Accepted:	7 July 2023

Original submission

First decision letter

MS ID#: DEVELOP/2023/201636

MS TITLE: Systematic analysis of the Frazzled receptor interactome establishes new regulators of axon guidance

AUTHORS: Yixin Zang and Greg Bashaw

I have now received the reports of three referees on your manuscript and I have reached a decision. The reports are appended below and you can access them online. For this, please go to and click on the 'Manuscripts with Decisions' queue in the Author Area.

As you will see, all the referees are enthusiastic about your work, but they also have significant criticisms and recommend a substantial revision of your manuscript before we can consider publication. In particular, they recommend that you validate the interaction of Fra with Lar, Rho-5 and CSN subunits by co-immunoprecipitation, as done for other candidate interactors. The experiments suggested in points 5 to 7 of Referee 2 would not significantly strengthen the conclusions of the manuscript and do not seem necessary. If you are able to revise the manuscript along the lines suggested, which may involve further experiments, I will be happy to receive a revised version of the manuscript. Your revised paper will be re-reviewed by one or more of the original referees, and acceptance of your manuscript will depend on your addressing satisfactorily the reviewers' major concerns. Please also note that Development will normally permit only one round of major revision. If it would be helpful, you are welcome to contact us to discuss your revision in greater detail. Please send us a point-by-point response indicating your plans for addressing the referees' comments, and we will look over this and provide further guidance.

Please attend to all of the reviewers' comments and ensure that you clearly highlight all changes made in the revised manuscript. Please avoid using 'Tracked changes' in Word files as these are lost in PDF conversion. I should be grateful if you would also provide a point-by-point response detailing how you have dealt with the points raised by the reviewers in the 'Response to Reviewers' box. If you do not agree with any of their criticisms or suggestions please explain clearly why this is so.

© 2023. Published by The Company of Biologists under the terms of the Creative Commons Attribution License (https://creativecommons.org/licenses/by/4.0/).

Reviewer 1

Advance summary and potential significance to field

This manuscript reports the unbiased identification of novel interacting partners of the netrin receptor Fra and tests the role of three of them, Lar, Rho-5 and members of the COP9 signalosome, in midline axon guidance. Using Fra affinity purification coupled to label free mass spectrometry, the authors identified 86 potential Fra interactors and confirmed four of these interactions by co-IP. They further demonstrate that the receptor Lar, the pseudoprotease Rho-5 and CSN subunits regulate midline axon crossing using loss-of-function approaches in a Fra sensitized genetic background. Since not much is known about how Fra/DCC signals in a Netrin-independent manner, this study makes a significant contribution to the field by uncovering novel potential regulators of this pathway. The Fra interactome described here is a valuable resource for the community and will likely prompt novel avenues of research in development.

Comments for the author

Overall, the study is well carried out, technically of high standard and very well written. It is appropriate for publication in Development provided that the authors address the few concerns detailed below:
1. The authors identified Fra, Rho-5 and CSN members as enriched proteins in the Fra interactome and validated these candidates by functional approaches in vivo.
However, functional approaches do not demonstrate an interaction with Fra, which the authors nicely confirmed by co-IP for four other proteins (LexB, Toll-7, Emb and Flo-2). The manuscript would gain in significance by also confirming the interaction between Fra and the proteins studied in vivo.
2. Chi-squared tests and Fisher's exact tests should be used for comparing categorical variables (percentages).

Minor points:
1. It would be nice to define what comm is in the first part of the results for readers who do not work on axon guidance.
2. Figures 1C and 1E show UAS-tau-myc-GFP while the text indicates the use of UAS-cd8-GFP. The UAS:cd8-GFP should also be mentioned in the Material and Methods section.
3. In Figure S2C, it seems that the y-axis should be “% of crossing EW segments”.
4. It would be informative to show pictures of single fra and lar mutants in Figure 5 since the authors are comparing double mutants to them.

Reviewer 2

Advance summary and potential significance to field

Frazzled is a conserved netrin receptor that is required for axon guidance, among many other developmental programs. Frazzled also functions independently of netrin to regulate gene transcription via its intracellular domain. In this manuscript Zang and Bashaw take two approaches to identify Fra interactors that regulate the Fra canonical and non-canonical pathways: a candidate approach based on other studies and an unbiased proteomic approach. Because of the fundamental, conserved functions of Fra signaling, the additional unknown Fra ligands that contribute to its functions, and its implications in disease, this work is relevant to a wide audience from various fields.

Comments for the author

Frazzled is a conserved netrin receptor that is required for axon guidance, among many other developmental programs. Frazzled also functions independently of netrin to regulate gene transcription via its intracellular domain. In this manuscript, Zang and Bashaw take two approaches to identify Fra interactors that regulate the Fra canonical and non-canonical pathways: a candidate approach based on other studies and an unbiased proteomic approach. Because of the fundamental, conserved functions of Fra signaling, the additional unknown Fra ligands that

© 2023. Published by The Company of Biologists under the terms of the Creative Commons Attribution License (https://creativecommons.org/licenses/by/4.0/).

contribute to its functions, and its implications in disease, this work is relevant to a wide audience from various fields.

Zang and Bashaw first demonstrate that signals from midline cells in the embryonic ventral nerve cord are required for comm expression in EW neurons. To identify new fly ligands for Fra, the authors tested a known Fra interactor from C. elegans but found that it likely does not function with Fra. The authors then turned to a proteomic approach – immunoprecipitation followed by mass spectrometry (IP-MS) – to identify novel, in vivo Fra interactors. A HA-tagged Fra was overexpressed pan-neuronally and embryos were collected. 85 candidates were identified, and several were tested for roles in Fra- dependent signaling: Lar, CSN subunits, and Rho-5. Although the data do not address whether the interactions are direct, the authors present compelling evidence for new Fra interactors that will guide future studies. However, before this manuscript is accepted, I discuss a few major and minor points below that should be addressed.

Major:

1. The authors confirmed some candidate Fra interactors (Emb, Flo2, Toll-7, and PlexinB) from their IP-MS in S2 cell co-IPs. However, when testing if candidates from their IP-MS function in Fra-mediated mechanisms, they tested a completely different set of candidates (Lar, CSN subunits, and Rho-5). The authors should at least test some of these candidates for Fra interaction in S2 cells.
2. In Figure 5, lar, fra double mutants are examined and the authors state that “these phenotypes are much more severe that what have been documented for fra mutants and lar mutants alone”. The authors should redo the single mutant experiments because the analyses/quantification can vary greatly from individual to individual. To draw meaningful comparisons between double and single mutants, the phenotypes should be scored in parallel.
3. Given that “the cytoplasmic domain of Fra is dispensable for receptor activity” (lines 506-507) but “Fra interactome is enriched for proteins with known intracellular localization” (lines 288-289), what does this say about the candidates identified in this study? Does the HA tag position on the intracellular position of the protein perhaps bias intracellular interactors? Please comment in the discussion.
4. Figure 5I-L: these images are used to argue for Lar and Fra function in “muscle targeting of motor axons”. However, no muscles are shown, no indication is given to show where the targeting defect is, and there is no quantification. The authors should update this figure if they want to argue that muscle targeting is affected by loss of lar and Fra.
5. Figure 7: Does the rho-5K03/+ background alone have an EW axon crossing defect? How about the homozygous rho-5K03? And the Rho-5 overexpression alone? Showing that these conditions also have axon crossing phenotypes would bolster their roles in axon guidance.
6. The authors present convincing data for novel Fra interactors and their roles in Fra-dependent EW axon pathfinding. Additionally, for some candidates, they show expression to suggest that Fra and the candidate function in the same cell type. To bolster this conclusion, the authors can use a egGAL4>UAS-FraΔC, UAS-candidate-RNAi background to knockdown candidate levels specifically in EW neurons.
7. Figure 6: Heterozygotes from CSN subunits are used to argue for the roles in Fra-dependent midline axon guidance. However, CSN subunit hets alone (without egGAL4>UAS-FraΔC) are not shown to properly compare the genotypes.

Minor:

1. “Here, we have shown that Rho-5 exhibits overlapping expression with Tace, as both are specifically enriched on the soma of ventral nerve cord neurons (Figure 7).” Figure 7 does not show Tace expression. This conclusion is based on a previous publication from the same lab showing that Tace is expressed in the ventral nerve cord. The authors should modify their language.
2. The figures that use red and green are not colorblind friendly, and although individual channels are shown in gray scale, the composite could be changed to magenta/green or another color combination that is colorblind friendly.
3. Please expand in the Methods on how stage 14 embryos were isolated in IP-MS experiments. This will help readers who may want to do a similar experiment for their favorite protein of interest.
4. “the overall protein abundance between experimental conditions is comparable as shown by the

© 2023. Published by The Company of Biologists under the terms of the Creative Commons Attribution License (https://creativecommons.org/licenses/by/4.0/).

distribution histogram (Figure S3B), suggesting that Fra-overexpression does note induce global changes in protein translations". Since the authors do not measure global protein levels, the protein counts observed could be due to bead saturation or limitations of protein isolation rather than Fra not impacting global protein expression.
5. Figure 2E: Very difficult to see the additional bands the authors suggest are in the experimental fraction. Maybe add asterisks where these bands are?
6. Figure 5K': "In lar, fra double mutants, we observed enlarged, irregularly shaped neurons with punctate membranes". What does this mean? Have similar phenotypes been reported for Lar or Fra? Also, in the figure, add arrows or asterisks to show exactly what the authors are referring to. Or maybe an enlarged zoomed in crop?
7. Is it known where the CSN subunits are expressed? Are they co-expressed with Fra? If antibodies or gene trap lines are available, the authors can explore this. In situ could also be performed similar to their comm RNA in situ experiments.

Reviewer 3

Advance summary and potential significance to field

Axons are attracted to the midline of the CNS by Netrins acting through Frazzled receptors and additional unidentified cues. The Bashaw group has previously shown that Frazzled responds to an unidentified ligand to induce Comm transcription. In this paper, Zang and Bashaw attempt to identify candidate ligands primarily using a mass spectrometry approach. A large number of interesting proteins were identified including receptors and trafficking proteins, but the ligand remains elusive. Some provocative hits were validated notably Lar and Rho-5. A candidate ligand Nolo/madd-4, identified in C. elegans was eliminated. The paper is well executed, and the dataset will be an extremely useful source of information for future experiments.

Comments for the author

The previous work on the localization of Lar ligands combined with the double mutant data in this paper should open new lines of investigation. The Lar protein subcellular localization pattern is stunning and it is surprising that it was not seen in previous reports. What is the source of the antibody?

The observed interaction with Robo1 is important and should be included in the abstract. It has not been previously reported in the fly and some questions exist about the vertebrate interaction.

Cite McIlroy et al (2013) for Toll-7 in the embryonic CNS given the discussion of DCC dependence pathways in the introduction.

The absence of NetA from the dataset is surprising and may be worth a comment.

First revision

Author response to reviewers' comments

We thank the reviewers for their thoughtful and constructive comments on our manuscript. Here we provide a point-by-point response to reviewers' comments, along with detailed experimental results to address the reviewer's questions and concerns, as described below in blue font. We believe that our revision has significantly strengthened the manuscript and hope the editor and reviewers will agree that our work is now suitable for publication in Development.

Reviewer 1 Advance Summary and Potential Significance to Field:
This manuscript reports the unbiased identification of novel interacting partners of the netrin receptor Fra and tests the role of three of them, Lar, Rho-5 and members of the COP9 signalosome,

© 2023. Published by The Company of Biologists under the terms of the Creative Commons Attribution License (https://creativecommons.org/licenses/by/4.0/).

in midline axon guidance. Using Fra affinity purification coupled to label free mass spectrometry, the authors identified 86 potential Fra interactors and confirmed four of these interactions by co-IP. They further demonstrate that the receptor Lar, the pseudoprotease Rho-5 and CSN subunits regulate midline axon crossing using loss-of-function approaches in a Fra sensitized genetic background. Since not much is known about how Fra/DCC signals in a Netrin- independent manner, this study makes a significant contribution to the field by uncovering novel potential regulators of this pathway. The Fra interactome described here is a valuable resource
for the community and will likely prompt novel avenues of research in development.

We appreciate the reviewer’s positive comments on our manuscript.

Reviewer 1 Comments for the Author:
Overall, the study is well carried out, technically of high standard and very well written. It is appropriate for publication in Development provided that the authors address the few concerns detailed below:

1. The authors identified Fra, Rho-5 and CSN members as enriched proteins in the Fra interactome and validated these candidates by functional approaches in vivo. However, functional approaches do not demonstrate an interaction with Fra, which the authors nicely confirmed by co-IP for four other proteins (LexB, Toll-7, Emb and Flo-2). The manuscript would gain in significance by also confirming the interaction between Fra and the proteins studied in vivo.

We appreciate this suggestion and agree with the reviewer that confirming the interaction between Fra and Lar, CSN subunits and Rho-5 will strengthen our conclusions. To address this comment, we cloned the coding sequences of Lar, CSN4 and Alien into expression plasmids, as fully sequenced cDNAs for these proteins can be easily obtained from the Drosophila Genomics Resource Center. We were able to confirm that Lar and CSN4 co-immunoprecipitate and physically interact with Fra, and that Alien shows non-specific binding which precludes further analysis of its association with Fra (Figure 5A and 6A). Unfortunately, we were unsuccessful in generating the cDNA and expression plasmid for Rho-5, so we were unable to test if Rho-5 interact with Fra by co-immunoprecipitation. However, we believe that based on the high fold change in the interactome data set (960.95) and the observation that both Fra and Rho-5 are highly expressed in the majority of neurons in the embryonic nerve cord (Figure 7), it is very likely that these two proteins will physically interact.

2. Chi-squared tests and Fisher's exact tests should be used for comparing categorical variables (percentages).

We appreciate this suggestion. We performed Chi-squared analysis on categorical variables in Figure S5, where the phenotypes in HRP labeled commissures fall into one of the three categories (“normal”, “thin” and “missing”). We performed student t-test or one-way ANOVA test on continuous variables in all the rest of our analysis. This is because the “percentage of non-crossing EW axons” is a continuous variable that can fall anywhere between 0% to 100%, rather than belonging to pre-set categories. We hope the reviewer agrees with our method of analysis.

Minor points:

1. It would be nice to define what comm is in the first part of the results for readers who do not work on axon guidance.

We appreciate this suggestion and we have added more information about Comm in the first result section.

2. Figures 1C and 1E show UAS-tau-myc-GFP while the text indicates the use of UAS-cd8-GFP. The UAS:cd8-GFP should also be mentioned in the Material and Methods section.

We appreciate the reviewer for pointing this out. We have replaced the UAS-cd8-GFP with UAS- tau-Myc-GFP.

3. In Figure S2C, it seems that the y-axis should be “% of crossing EW segments”.
We appreciate the reviewer for pointing this out. We have modified Figure S2C.

© 2023. Published by The Company of Biologists under the terms of the Creative Commons Attribution License (https://creativecommons.org/licenses/by/4.0/).

4. It would be informative to show pictures of single fra and lar mutants in Figure 5 since the authors are comparing double mutants to them.

We thank the reviewer for this valuable suggestion. When addressing this comment, we observed that *lar*$^{13.2}$ mutants show the same commissure formation and cell morphology defects as the *fra*^{3},*lar*$^{13.2}$ double mutants. We believe this phenotype results from a background mutation that is unrelated to *lar,* as compound heterozygotes with two different *lar* alleles do not show these phenotypes. To ensure that this background mutation did not arise from the *lar*$^{13.2}$ fly line maintained in our lab, we have repeated the experiment with a new *lar*$^{13.2}$ line ordered directly from the Bloomington Drosophila Stock Center and observed the same phenotypes. Due to this background mutation, we are unable to test genetic interaction between *fra* and *lar* (Figure S5). The *lar*$^{13.2}$ allele is one of the most frequently used alleles of lar, yet this phenotype has not been documented before. Thus, we decided to include this observation in the manuscript as a supplementary figure (Figure S5) despite the fact that we cannot conclude from this experiment whether *fra* and *lar* function in the same or parallel genetic pathways.

Because the background mutation in *lar*$^{13.2}$ mutants severely affects midline crossing, we decided to remove the data describing the increased EW axon non-crossing phenotype observed in homozygous *lar*$^{13.2}$ mutant using the FraDC genetic background. We elect to keep the data describing the removal of one copy of *lar* using *lar*$^{13.2}$ (Figure 5F to FH), and supplement it by testing several additional *lar* mutant alleles (Figure 5I to 5N). Our results indicate that Lar function in midline crossing of commissural axons, but the precise mechanism remains to be explored.

Reviewer 2 Advance Summary and Potential Significance to Field

Frazzled is a conserved netrin receptor that is required for axon guidance, among many other developmental programs. Frazzled also functions independently of netrin to regulate gene transcription via its intracellular domain. In this manuscript, Zang and Bashaw take two approaches to identify Fra interactors that regulate the Fra canonical and non-canonical pathways: a candidate approach based on other studies and an unbiased proteomic approach. Because of the fundamental, conserved functions of Fra signaling, the additional unknown Fra ligands that contribute to its functions, and its implications in disease, this work is relevant to a wide audience from various fields.

We appreciate the reviewer’s positive comments on our manuscript.

Reviewer 2 Comments for the author
Zang and Bashaw first demonstrate that signals from midline cells in the embryonic ventral nerve cord are required for comm expression in EW neurons. To identify new fly ligands for Fra, the authors tested a known Fra interactor from C. elegans but found that it likely does not function with Fra. The authors then turned to a proteomic approach - immunoprecipitation followed by mass spectrometry (IP-MS) - to identify novel, in vivo Fra interactors. A HA-tagged Fra was overexpressed pan-neuronally and embryos were collected. 85 candidates were identified, and several were tested for roles in Fra-dependent signaling: Lar, CSN subunits, and Rho-5. Although the data do not address whether the interactions are direct, the authors present compelling evidence for new Fra interactors that will guide future studies. However, before this manuscript is accepted, I discuss a few major and minor points below that should be addressed.

Major:
1. The authors confirmed some candidate Fra interactors (Emb, Flo2, Toll-7, and PlexinB) from their IP-MS in S2 cell co-IPs. However, when testing if candidates from their IP-MS function in Fra-mediated mechanisms, they tested a completely different set of candidates (Lar, CSN subunits, and Rho-5). The authors should at least test some of these candidates for Fra interaction in S2 cells.

We appreciate this suggestion and have included the experiments suggested by the reviewer in Figure 5A and Figure 6A. For details, please refer to our response to major point #1 from review 1.

© 2023. Published by The Company of Biologists under the terms of the Creative Commons Attribution License (https://creativecommons.org/licenses/by/4.0/).

2. In Figure 5, lar, fra double mutants are examined and the authors state that these phenotypes are much more severe that what have been documented for fra mutants and lar mutants alone". The authors should redo the single mutant experiments because the analyses/quantification can vary greatly from individual to individual. To draw meaningful comparisons between double and single mutants, the phenotypes should be scored in parallel.

We agree with the reviewer and thank the reviewer for this valuable comment. We have re-analyzed the single mutants and have included this experiment in Figure S5. For details, please refer to our response to minor point #4 from review 1.

3. Given that the cytoplasmic domain of Fra is dispensable for receptor activity (lines 506-507) but Fra interactome is enriched for proteins with known intracellular localization (lines 288-289), what does this say about the candidates identified in this study? Does the HA tag position on the intracellular position of the protein perhaps bias intracellular interactors? Please comment in the discussion.

We appreciate this comment but want to clarify that in the text we meant that only "the tyrosine phosphorylation in the cytoplasmic domain of Fra" is dispensable for receptor activity, rather than the entire cytoplasmic domain of Fra. The cytoplasmic domains of Fra are essential for Fra's signaling properties and function by either recruiting downstream signaling effectors or acting as a transcriptional regulator. Thus, we expect to identify many intracellular proteins in our interactome as potential effectors or regulators of the Fra signaling pathway.

4. Figure 5I-L: these images are used to argue for Lar and Fra function in muscle targeting of motor axons. However, no muscles are shown, no indication is given to show where the targeting defect is, and there is no quantification. The authors should update this figure if they want to argue that muscle targeting is affected by loss of lar and Fra.

We thank the reviewer for this suggestion. Because we have observed an unexpected effect from a background mutation in the $lar^{13.2}$ mutants (Figure S5), we have decided to remove the part about muscle targeting of motor axons, as this phenotype could either result from the loss of *lar* or the loss of another gene.

Minor:
1. Here, we have shown that Rho-5 exhibits overlapping expression with Tace, as both are specifically enriched on the soma of ventral nerve cord neurons (Figure 7). Figure 7 does not show Tace expression. This conclusion is based on a previous publication from the same lab showing that Tace is expressed in the ventral nerve cord. The authors should modify their language.

We appreciate the reviewer for pointing this out. We have modified this part as suggested by the reviewer.

2. The figures that use red and green are not colorblind friendly, and although individual channels are shown in gray scale, the composite could be changed to magenta/green or another color combination that is colorblind friendly.

We appreciate the reviewer for pointing this out. We have modified the figures as suggested by the reviewer.

3. Please expand in the Methods on how stage 14 embryos were isolated in IP-MS experiments. This will help readers who may want to do a similar experiment for their favorite protein of interest.

We appreciate this comment but want to clarify that we used embryos collected in a 24-hour collection for the IP-MS experiments, which includes embryos from stage 1 to stage 17. We have emphasized this information in the method section.

4. The overall protein abundance between experimental conditions is comparable as shown by the distribution histogram (Figure S3B), suggesting that Fra-overexpression does note induce global

© 2023. Published by The Company of Biologists under the terms of the Creative Commons Attribution License (https://creativecommons.org/licenses/by/4.0/).

changes in protein translations. Since the authors do not measure global protein levels, the protein counts observed could be due to bead saturation or limitations of protein isolation rather than Fra not impacting global protein expression.

We appreciate this comment and agree with the reviewer that these are possible explanations of our data. However, to reduce the likelihood of these possibilities, we added a huge excess of beads and antibodies in our experiments. We have added this information in the method section.

5. Figure 2E: Very difficult to see the additional bands the authors suggest are in the experimental fraction. Maybe add asterisks where these bands are?

We appreciate the reviewer for pointing this out. We have modified Figure 2E as suggested by the reviewer.

6. Figure 5K': In lar, fra double mutants, we observed enlarged, irregularly shaped neurons with punctate membranes. What does this mean? Have similar phenotypes been reported for Lar or Fra? Also, in the figure, add arrows or asterisks to show exactly what the authors are referring to. Or maybe an enlarged zoomed in crop?

We thank the reviewer for this comment. The phenotype referred to by the reviewer is the noticeable difference in cell morphology in Figures S5B' and S5F', as compared to Figures S5A', S5C' toS5E'. We have modified the text and the legend of Figure S5, and added enlarged panels (Figures S5A'', S5B'' and S5F''). We believe this phenotype also results from the background mutation, because the same phenotype is not observed in the compound heterozygotes.

7. Is it known where the CSN subunits are expressed? Are they co-expressed with Fra? If antibodies or gene trap lines are available, the authors can explore this. In situ could also be performed similar to their comm RNA in situ experiments.

We thank the reviewer for this comment. The expression of CSN subunits in embryos has not been examined. Data from the BDGP expression data set suggests that CSN4 is ubiquitously expressed. Unfortunately, no antibodies, gene trap lines or RNA *in situ* probes are publicly available.

Reviewer 3 Advance Summary and Potential Significance to Field:

Axons are attracted to the midline of the CNS by Netrins acting through Frazzled receptors and additional unidentified cues. The Bashaw group has previously shown that Frazzled responds to an unidentified ligand to induce Comm transcription. In this paper, Zang and Bashaw attempt to identify candidate ligands primarily using a mass spectrometry approach. A large number of interesting proteins were identified including receptors and trafficking proteins, but the ligand remains elusive. Some provocative hits were validated, notably Lar and Rho-5. A candidate ligand Nolo/madd-4, identified in C. elegans was eliminated. The paper is well executed, and the dataset will be an extremely useful source of information for future experiments.

We appreciate the reviewer's positive comments on our manuscript.

Reviewer 3 Comments for the Author:
The previous work on the localization of Lar ligands combined with the double mutant data in this paper should open new lines of investigation. The Lar protein subcellular localization pattern is stunning and it is surprising that it was not seen in previous reports. What is the source of the antibody?

We appreciate this comment. The antibody is available through Developmental Studies Hybridoma Bank (Cat#9D82B3). It has been shown that Lar protein is selective localized to longitudinal tracks in late stage 16 embryos by Sun et al. (2000), but we believe a time course of Lar expression across different developmental stages has not been reported previously.

The observed interaction with Robo1 is important and should be included in the abstract. It has not been previously reported in the fly and some questions exist about the vertebrate interaction.

© 2023. Published by The Company of Biologists under the terms of the Creative Commons Attribution License (https://creativecommons.org/licenses/by/4.0/).

We appreciate the reviewer for pointing this out. We have modified the abstract as suggested by the reviewer.

Cite McIlroy et al (2013) for Toll-7 in the embryonic CNS given the discussion of DCC dependence pathways in the introduction.

We thank the reviewer for bringing this important citation to our attention. We have cited this work in our manuscript.

The absence of NetA from the dataset is surprising and may be worth a comment.

We appreciate this comment and agree with the reviewer that the presence of NetB but not NetA in our Fra interactome is surprising. Because of the high degree of sequence homology between NetB and NetA, we suspect that trypsinizing NetA and NetB will lead to the formation of short peptides that are very similar. For reasons unknown to us, these peptides might be preferentially mapped to NetB. Alternatively, the interaction between a secreted ligand and the extracellular region of its receptor might be too weak and transient to be reliably captured by our method. In the future, methods that enrich for membrane proteins or methods that cross-link interacting proteins might be better suited to identify interactions between ligands and receptors.

Second decision letter

MS ID#: DEVELOP/2023/201636

MS TITLE: Systematic analysis of the Frazzled receptor interactome establishes new regulators of axon guidance

AUTHORS: Yixin Zang and Greg Bashaw

I have now received the reports of two of the referees who reviewed the earlier version of your manuscript and I have reached a decision. The referees' comments are appended below, or you can access them online: please go to BenchPress and click on the 'Manuscripts with Decisions' queue in the Author Area.

The overall evaluation is positive and we would like to publish a revised manuscript in Development, provided that you satisfactorily address the remaining comments of referee 2. Please attend to all of the reviewer's comments in your revised manuscript and detail them in your point-by-point response. If you do not agree with any of their criticisms or suggestions explain clearly why this is so.

Reviewer 1

Advance summary and potential significance to field

This manuscript reports the unbiased identification of novel interacting partners of the netrin receptor Fra and tests the role of three of them, Lar, Rho-5 and members of the COP9 signalosome, in midline axon guidance in vivo. Using Fra affinity purification coupled to label free mass spectrometry, the authors identified 86 potential Fra interactors and confirmed several of these interactions by co-IP. They further demonstrate that the receptor Lar, the pseudoprotease Rho-5 and CSN subunits regulate midline axon crossing using loss-of-function approaches in a Fra sensitized genetic background. Since not much is known about how Fra/DCC signals in a Netrin-independent manner, this study makes a significant contribution to the field by uncovering novel potential regulators of this pathway. The Fra interactome described here is a valuable resource for the community and will likely prompt novel avenues of research in development.

© 2023. Published by The Company of Biologists under the terms of the Creative Commons Attribution License (https://creativecommons.org/licenses/by/4.0/).

Comments for the author

The study is well carried out, technically of high standard and very well written.
The authors have addressed all my concerns. Their revised manuscript is now appropriate for publication and will be of high interest to readers of Development.

Reviewer 2

Advance summary and potential significance to field

Frazzled is a conserved netrin receptor required for axon guidance, among many other developmental programs. Frazzled also functions independently of netrin to regulate gene transcription via its intracellular domain. In this manuscript, Zang and Bashaw take two approaches to identify Fra interactors that regulate the Fra canonical and non-canonical pathways: a candidate approach based on other studies and an unbiased proteomic approach. Because of the fundamental, conserved functions of Fra signaling, the additional unknown Fra ligands that contribute to its functions, and its implications in disease, this work is relevant to a wide audience from various fields.

Comments for the author

In this revision manuscript, Zang and Bashaw have refined their study identifying potential Fra interactors and have augmented the work with new experiments and analyses.
There are a couple of minor comments below that should be addressed. However these comments are not extensive and therefore do not require any additional round of peer review.
- "...Fra interacting proteins in Drosophila revealed that they are most abundantly expressed in stage 13-16 embryos in the larval CNS (Figures 3A and B)". Maybe I am readying Figure 3A incorrectly, but it seems like stage 4-6 embryos express more interacting proteins? Also, why are the stages in the X axis ordered seemingly randomly? Why not order from youngest to oldest embryos?
- Figure 5B'-E': This is the grey scale/BW version of 5B-E. This is not LAR as indicated. Please update.
- "This result indicates that different truncations or manipulations of the Lar protein have distinct impact on the midline axon guidance, suggesting that Lar is important for this process." But when testing the different lar mutants, the authors find that lar13.2 shows a severe disruption of commissure formation
(Figures S5A, SB', S5G) that is absent in other lar homozygous mutants. The authors suggest that the phenotype may be due to a background mutation. While this is possible, then why is the fra,lar13.2 double mutant phenotype significantly worse than lar13.2 homozygous mutants?

Second revision

Author response to reviewers' comments

Response to reviewers

Reviewer comment:
There are a couple of minor comments below that should be addressed. However, these comments are not extensive and therefore do not require any additional round of peer review.

- "...Fra interacting proteins in Drosophila revealed that they are most abundantly expressed in stage 13-16 embryos in the larval CNS (Figures 3A and B)". Maybe I am readying Figure 3A incorrectly, but it seems like stage 4-6 embryos express more interacting proteins? Also, why are the stages in the X axis ordered seemingly randomly? Why not order from youngest to oldest embryos?

© 2023. Published by The Company of Biologists under the terms of the Creative Commons Attribution License (https://creativecommons.org/licenses/by/4.0/).

Author Response: We have modified the text to describe the temporal expression pattern of candidate genes more clearly. This section now reads: ‘As expected, Gene Ontology (GO) analysis of the spatial and temporal expression profiles of candidate Fra interacting proteins in Drosophila revealed that they are expressed throughout embryogenesis including stage 13-16 and are enriched in the Larval CNS (Figures 3A and B).’

We have rearranged Figure 3A as suggested.

Reviewer comment:- Figure 5B’-E’: This is the grey scale/BW version of 5B-E. This is not LAR as indicated. Please update.

Author Response: The figure is correctly labeled. The grey scale images in 5 B-E’ shows only Lar staining, while 5 B-E shows HRP in magenta and Lar in green.

Reviewer comment: - “This result indicates that different truncations or manipulations of the Lar protein have distinct impact on the midline axon guidance, suggesting that Lar is important for this process.” But when testing the different lar mutants, the authors find that lar13.2 shows a severe disruption of commissure formation (Figures S5A, SB’, S5G) that is absent in other lar homozygous mutants. The authors suggest that the phenotype may be due to a background mutation. While this is possible, then why is the fra,lar13.2 double mutant phenotype significantly worse than lar13.2 homozygous mutants?

Author Response: Quantitative comparison between lar13.2 homozygous mutants and lar13.2, fra double mutants reveals that these phenotypes are not statistically different from each other, as indicated in Figure S5G.

Third decision letter

MS ID#: DEVELOP/2023/201636

MS TITLE: Systematic analysis of the Frazzled receptor interactome establishes new regulators of axon guidance

AUTHORS: Yixin Zang and Greg Bashaw
ARTICLE TYPE: Techniques and Resources Article

I am delighted to tell you that your manuscript has been accepted for publication in Development, pending our standard ethics checks.

© 2023. Published by The Company of Biologists under the terms of the Creative Commons Attribution License (https://creativecommons.org/licenses/by/4.0/).
